# Intramolecular Carbene C-H Insertion Reactions of 2-Diazo-2-sulfamoylacetamides

**DOI:** 10.3390/molecules24142628

**Published:** 2019-07-19

**Authors:** Chuqiang Que, Peipei Huang, Zhanhui Yang, Ning Chen, Jiaxi Xu

**Affiliations:** State Key Laboratory of Chemical Resource Engineering, Department of Organic Chemistry, College of Chemistry, Beijing University of Chemical Technology, Beijing 100029, China

**Keywords:** amide, carbene insertion, C-H insertion, competitive reaction, diazo compound, sulfonamide, sultam

## Abstract

The intramolecular C-H insertions of carbenes derived from 2-diazo-2-sulfamoylacetamides were studied. 2-Diazo-2-sulfamoylacetamides were first prepared from chloroacetyl chloride and secondary amines through acylation followed by sequential treatments with sodium sulfite, phosphorus oxychloride, secondary amines, and 4-nitrobenzenesulfonyl azide. The results indicate that: (1) 2-diazo-*N,N*-dimethyl-2-(*N,N*-diphenylsulfamoyl)acetamide can take the formal aromatic 1,5-C-H insertion in its *N*-phenylsulfonamide moiety to afford the corresponding 1,3-dihydrobenzo[*c*]isothiazole-3-carboxamide 2,2-dioxide derivative; (2) no aliphatic C-H insertions occur for 2-diazo-2-(*N,N*-dialkylsulfamoyl)acetamides; and (3) for 2-diazo-*N*-phenyl-2-(*N*-phenylsulfamoyl)acetamides, the formal aromatic 1,5-C-H insertion in the *N*-phenylacetamide moiety is favorable to afford the corresponding 3-sulfamoylindolin-2-one derivatives as sole or major products. The intramolecular competitive aromatic 1,5-C-H insertion reactions of 2-diazo-2-sulfamoylacetamides with aryl groups on both amide and sulfonamide groups reveal that the *N*-aryl substituents on acetamide are more active than those on sulfonamide. The chemoselectivity is controlled by electronic effect of the aryl group.

## 1. Introduction

**Scheme 1:**
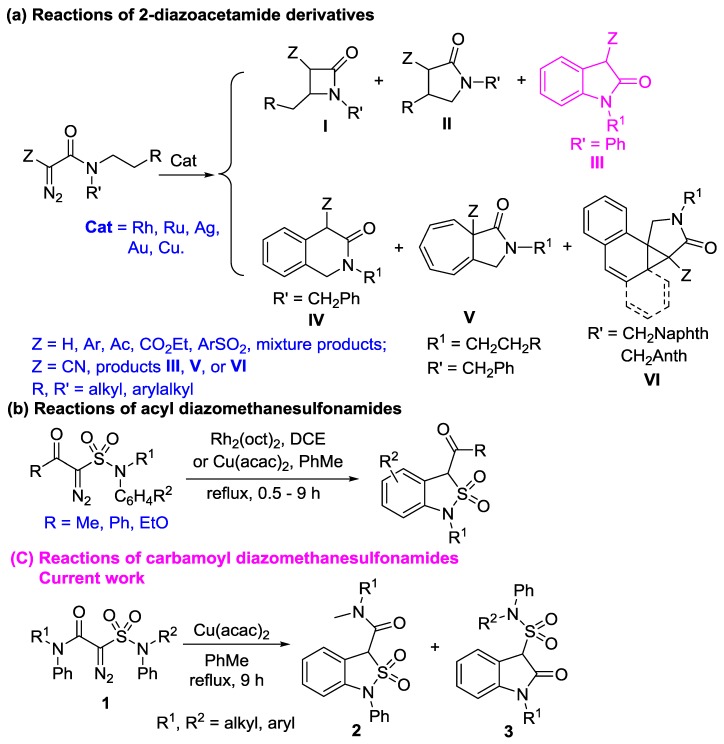
Intramolecular carbene C-H insertion reactions of diazoacetamide derivatives and acyl diazomethanesulfonamides.

The transition-metal-catalyzed intramolecularly aliphatic and aromatic C-H insertions of diazo amides provide important transformations to efficiently synthesize nitrogen-containing heterocycles with the new C-C bond formation [1,2,3,4]. The reactivity of diazoamides has been well studied (Scheme 1a), affording β-lactams (**I**) [5,6,7,8,9], γ-lactams (**II**) [5,6,7,8,9], indolin-2-ones (**III**) [10,11,12,13,14,15], 1,4-dihydroisoquinolin-3(2*H*)-ones (**IV**), Buchner products (**V**), and cyclopropanation products (**VI**) [16,17,18,19,20,21] (Scheme 1a) and a series of exquisite reactions has been established to construct complex and useful structures, such as biological and natural products [22,23].

Recently, we put much attention to the reactivity of different diazosulfonamides (Scheme 1b) [24,25], and realized the rhodium- and copper-catalyzed intramolecular aromatic 1,5-C-H insertion of *N,N*-diaryl diazomethanesulfonamides with the ethoxycarbonyl group (ethyl 2-(*N,N*-diarylsulfamoyl)-2-diazoacetates) to prepare ethyl 1-aryl-1,3-dihydrobenzo[*c*]isothiazole-3-carboxylate 2,2-dioxides (*N*-aryl-3-ethoxycarbonyl benzo-γ-sultams) (R = OEt, Scheme 1b) [24]. We also achieved the copper-catalyzed intramolecularly aromatic 1,5-C-H insertion of *N*-alkyl-*N*-aryl diazomethanesulfonamides **1** with the acetyl and benzoyl groups to synthesize 1-alkyl-3-acetyl/benzoyl-2,2-dioxido-1,3-dihydrobenzo[*c*]isothiazoles (R = Me, Ph, Scheme 1b) [25]. For the *N*-alkyldiazomethanesulfonamides with acetyl, benzoyl, and ethoxycarbonyl groups, the aliphatic C-H insertions, including 1,4-, 1,5-, and 1,6-insertions, of the *N*-alkyl groups on the sulfonamide moiety were not observed [24,25]. Compared with diazoacetamides, only limited investigations on the reactivity and chemoselectivity on diazosulfonamides were conducted [24,25]. 2-Diazo-2-sulfamoylacetamides are also carbamoyl diazomethanesulfonamides from the viewpoint of sulfonamides. They are diazomethanes with both carbamoyl and sulfamoyl groups. The following is not clear to date: (1) Do aromatic 1,5-C-H insertions of carbamoyl diazomethanesulfonamides take place on their *N*-phenyl group on sulfonamide? (2) Do aliphatic C-H insertions occur in 2-diazo-2-(*N,N*-dialkylsulfamoyl)acetamides? (3) If 2-diazo-2-sulfamoylacetamides have two aryl groups on different nitrogen atoms of their amide and sulfonamide groups, what is the chemoselectivity in their intramolecular competitive aromatic 1,5-C-H insertion reactions? Herein, we report our results on the intramolecular carbene C-H insertion reactions of 2-diazo-2-sulfamoylacetamides with different alkyl and aryl substituents on the nitrogen atoms of the amide and sulfonamide groups. 

## 2. Results and Discussion

### 2.1. Synthesis of 2-Diazo-2-sulfamoylacetamides ***1***

**Scheme 2:**
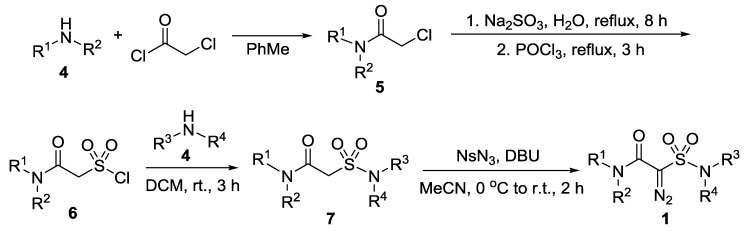
Synthesis of 2-diazo-2-sulfamoylacetamides.

Because the carbene C-H insertion of carbamoyl diazomethanesulfonamides has not been explored previously, to confirm whether *N*-aryl-carbamoyl-diazomethanesulfonamides can take the aromatic 1,5-C-H insertion in their *N*-arylsulfonamide moiety, we first prepared *N*-methyl-*N*-phenyl and *N,N*-diphenyl carbamoyl-diazomethanesulfonamides **1a** and **1b**. The reaction of 2-chloroacetyl chloride and dimethylammonium chloride (**4a**∙HCl) in the presence of triethylamine in dichloromethane afforded 2-chloro-*N,N*-dimethylacetamide (**5a**) in 90% yield. 2-Chloroacetamide **5a** was treated with sodium sulfite in water three times to give rise to solid sodium carbamoylmethanesulfonate after removal of water. The mixture of dry crude sodium sulfonate and phosphorus oxychloride was refluxed for 3 h to afford the corresponding sulfonyl chloride **6a**, which further reacted with *N*-methylaniline (**4b**) and diphenylamine (**4c**), respectively. However, only diphenylamine (**4c**) gave rise to sulfamoylacetamide **7b** in a low yield of 16%. It is somewhat strange that *N,N*-dimethylcarbamoylmethanesulfonyl chloride (**6a**) failed to react with *N*-methylaniline (**4b**) (Scheme 2). To verify the results, we attempted the reaction several times, but all failed to afford sulfamoylacetamide **7a** (Table 1, entry a).

The reactions of 2-chloroacetyl chloride and secondary amines **4b** and **4c** produced tertiary 2-chloroacetamides **5c** and **5d** in excellent yields. 2-Chloroacetamides **5c**,**d** were treated with sodium sulfite in water three times to give rise to solid sodium carbamoylmethanesulfonates after removal of water. The mixtures of dry crude sodium sulfonates and phosphorus oxychloride were refluxed for 3 h to afford the corresponding sulfonyl chlorides **6c**,**d**. To evaluate aliphatic C-H insertions of 2-diazo-2-(*N,N*-dialkylsulfamoyl)acetamides, sulfonyl chlorides **6c**,**d** were reacted with *N*-butylbenzylamine (**4d**) to yield 2-(*N*-benzyl-*N*-butylsulfamoyl)-2-diazoacetamides **7c**,**d** in satisfactory yields (Table 1, entries c and d). To investigate the intramolecular competitive aromatic 1,5-C-H insertion of *N*-aryl-2-(*N*-arylsulfamoyl)-2-diazoacetamides, sulfonyl chlorides **6c,d** were further reacted with another secondary amines **4b**,**c** to yield sulfamoylacetamides **7e**–**h** in 30–88% yields (Table 1, entries e–h). Sulfamoylacetamides **7** were converted into 2-diazosulfamoylacetamides **1** through the diazo transformation reaction with 4-nitrobenzenesulfonyl azide (NsN_3_) in acetonitrile with DBU as base (Scheme 2).

Although the reaction of *N,N*-diphenylcarbamoylmethanesulfonyl chloride (**6d**) and diphenylamine (**4c**) worked well (Table 1, entry h), when we tried to react it with di(4-methoxyphenyl)amine (**4e**), di(4-methylphenyl)amine (**4f**), di(4-chlorophenyl)amine (**4g**), and di(4-bromophenyl)amine (**4h**) to prepare different 2-(*N,N*-diarylsulfamoyl)-2-diazo-*N,N*-diphenylacetamides **1i**–**l** with different electron density phenyl groups on the nitrogen of the sulfonamide group, no reaction occurred or only trace amounts of the corresponding 2-diazosulfamoylacetamides were observed.

### 2.2. Intramolecular Carbene C-H Insertion Reactions of 2-Diazo-2-sulfamoylacetamides ***1***

The reaction of 2-diazo-*N,N*-dimethyl-2-(*N,N*-diphenylsulfamoyl)acetamide (**1b**) was applied to optimize the reaction conditions. The results indicate that 2-diazo-2-sulfamoylacetamide **1b** in refluxing toluene under stirring and the catalysis of Cu(acac)_2_ for 9 h is the optimal condition, similar to a previous study [25], affording *N,N*-dimethyl-1-phenyl-1,3-dihydrobenzo[*c*]isothiazole-3-carboxamide 2,2-dioxide (**2b**) in 90% yield (Table 2, entry 1). The results show that *N*-aryl-diazomethanesulfonamides with carbamoyl substituents are suitable substrates for the intramolecular aromatic 1,5-C-H insertion on the aryl group of sulfamide.

To further examine aliphatic C-H insertion of 2-diazo-2-sulfamoylacetamides, both 2-diazo-2-(*N*-benzyl-*N*-butylsulfamoyl)-*N*-methyl-*N*-phenylacetamide (**1c**) and 2-diazo-2-(*N*-benzyl-*N*-butylsulfamoyl)-*N*,*N*-diphenylacetamide (**1d**) were conducted under the optimal reaction conditions, affording 3-(*N*-benzyl-*N*-butylsulfamoyl)-1-methylindolin-2-one (**3c**) and 3-(*N*-benzyl-*N*-butylsulfamoyl)-1-phenylindolin-2-one (**3d**) chemospecifically in 76% and 85% yields, respectively (Table 2, entries 2 and 3). Similar to acyl diazomethanesulfonamides [24,25], aliphatic 1,4-, 1,5-, and 1,6-C-H insertions in the butyl group; aromatic 1,6-C-H insertion; Buchner reaction; and cyclopropanation on the benzene ring in the benzyl group did not occur, although diazosulfones and diazosulfonates favored aliphatic insertions [26,27,28,29,30,31,32].

Although the aliphatic carbene 1,4-C-H insertion of diazoamides generally occurred and was sensitive to the acyl groups [1,2,3,4,5,6,7,8,9,10,11,12,13,14,15], it appeared quite different in our attempted acyl diazo-*N*-methylmethanesulfonamides [25] and 2-diazo-2-(*N*-benzyl-*N*-butylsulfamoyl)acetamides **1c**,**d**, possibly because of the subtle changes in conformation or the bond length of diazomethanesulfonamides, as Doyle and coworkers mentioned [3].

To investigate the intramolecular competitive aromatic 1,5-C-H insertion reaction, four representative *N*-aryl-2-diazo-2-(*N*-arylsulfamoyl)acetamides **1e**–**h** with two, three, or four *N*-phenyl groups, respectively, were conducted under the optimal conditions. The results are presented in Table 2. 2-Diazo-*N*-methyl-*N*-phenyl-2-(*N*-methyl-*N*-phenylsulfamoyl)acetamide (**1e**) gave rise to chemospecific 1-methyl-3-(*N*-methyl-*N*-phenylsulfamoyl)indolin-2-one (**3e**) only in 53% yield (Table 2, entry 4). The structure was assigned by comparing the IR absorbing wave number of the C=O group in the amide moiety in **1e** (1658 cm**^−^**^1^), **2b** (1656 cm**^−^**^1^), and **3e** (1720 cm**^−^**^1^) (γ-lactam). However, 2-diazo-*N*-methyl-*N*-phenyl-2-(*N,N*-diphenylsulfamoyl)acetamide (**1f**) yielded both *N*-methyl-*N*,1-diphenyl-1,3-dihydrobenzo[*c*]isothiazole-3-carboxamide 2,2-dioxide (**2f**) in 15% yield and 1-methyl-3-(*N,N*-diphenylsulfamoyl)indolin-2-one (**3f**) in 44% yield, favoring the aromatic 1,5-C-H insertion in the *N*-phenyl amide moiety (Table 2, entry 5). Both 2-diazo-2-(*N*-methyl-*N*-phenylsulfamoyl)-*N*,*N*-diphenylacetamide (**1g**) and 2-diazo-*N*,*N*-diphenyl-2-(*N,N*-diphenylsulfamoyl)acetamide (**1h**) gave rise to chemospecific 3-(*N*-methyl-*N*-phenylsulfamoyl)-1-phenylindolin-2-one (**3g**) and 1-phenyl-3-(*N,N*-diphenylsulfamoyl)indolin-2-one (**3h**) in similar yields of 52% and 48%, respectively (Table 2, entries 6 and 7). No corresponding 1,3-dihydrobenzo[*c*]isothiazole-3-carboxamide 2,2-dioxide was observed in the reaction mixture in each of cases.

### 2.3. Mechanism on the Aromatic 1,5-C-H Insertion

**Scheme 3:**
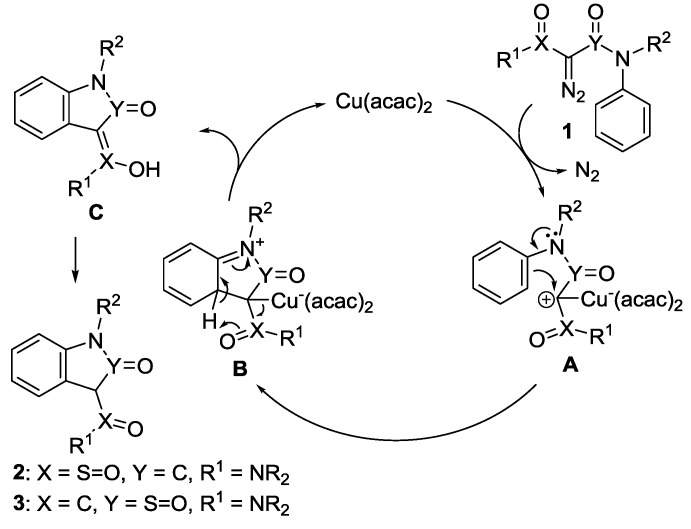
Proposed mechanism for the intramolecular aromatic 1,5-C-H insertion of diazosulfamoylacetamides **1**.

According to previous publications [1,2,3,4,14], considering the mechanism of the intramolecular aromatic 1,5-C-H insertion, it is proposed as follows. Diazosulfamoylacetamides **1** react with Cu(acac)_2_ to generate Cu-carbon ylides **A**, of which the carbocation electrophilically attacks the benzene ring in the *N*-phenyl group in the amide moiety (in the sulfonamide moiety only for 2-diazo-*N,N*-dialkyl(*N*-arylsulfamoyl)acetamides) to yield cyclized intermediates **B** (Frediel–Craft alkylation). After releasing Cu(acac)_2_ catalyst and the proton transfer, the intermediates **B** convert to aromatic 1,5-inserted enolic intermediates **C**, which tautomerize into final products **2** and/or **3** (Scheme 3).

### 2.4. Rationale on the Chemoselectivity in the Aromatic 1,5-C-H Insertions of N-aryl-2-diazo-2-(N-arylsulfamoyl)acetamides

2-Diazo-*N*-phenyl-2-(*N*-phenylsulfamoyl)acetamides **1e**–**h** generally predominantly reacted on the phenyl group of *N*-phenylacetamides, giving rise to 3-sulfamoylindolin-2-ones **3e**–**h** as major or exclusive products. Only 2-diazo-*N*-methyl-*N*-phenyl-2-(*N,N*-diphenylsulfamoyl)acetamide (**1f**) generated 1,3-dihydrobenzo[*c*]isothiazole-3-carboxamide 2,2-dioxide **2f** as a minor product at the same time.

On the basis of Hammett constants [33], methanesulfonyl (σ = 0.73) is a stronger electron-withdrawing group than acetyl (σ = 0.47). It should be reasonable to rationalize that the phenyl group of *N*-phenylamides is generally electron-richer than that of *N*-phenylsulfonamides, favoring the electrophilic attack of Cu-carbon ylides to the phenyl group of amides, showing higher reactivity of *N*-phenylacetamides. The chemoselectivity is attributed to the different electronic effects of methanesulfonamido and acetamido groups when amides and sulfonamides possess similar steric hindrance. For example, the O=C-N(Me)-Ph and (O=)_2_S-N(Me)-Ph conjugative systems in substrate **1e** can exist in the same planes or almost same planes. Thus, the electron-withdrawing conjugative effect of the methanesulfonamido group is stronger than that of the acetamido group, resulting in lower electron density of the phenyl group on sulfonamide. Thus, the aromatic 1,5-C-H insertion occurs on the phenyl group of acetamide. In *N*,*N*-diphenyl derivative **1h**, both O=C-N(Ph)-Ph and O=S-N(Ph)-Ph conjugative systems cannot exist in the same planes due to steric hindrance of two phenyl groups. The phenyl group of acetamide is electron-richer than that of sulfonamide as well in **1h**. The phenyl group of O=C-N(Ph)-Ph is absolutely electron-richer than that of (O=)_2_S-N(Me)-Ph in substrate **1g** because the former is not in the same plane due to steric hindrance of two phenyl groups, while the latter is in the same plane. Similarly, the aromatic 1,5-C-H insertion of both substrate **1g** and **1h** occurs on the electron-rich phenyl group of acetamide. However, for 2-diazo-*N*-methyl-*N*-phenyl-2-(*N,N*-diphenylsulfamoyl)acetamide (**1f**), the (O=)_2_S-N(Ph)-Ph conjugative system is non-planar, showing weak electron-withdrawing conjugative effect due to steric hindrance of two phenyl groups, while the near planar O=C-N(Me)-Ph conjugative system shows strong electron-withdrawing conjugative effect to decrease the electron density of the phenyl group on the *N*-phenylacetamide, resulting in closer electron-densities of these two phenyl groups on both acetamide and sulfonamide. This is the reason 2-diazo-*N*-methyl-*N*-phenyl-2-(*N,N*-diphenylsulfamoyl)acetamide (**1f**) chemoselectively generated both 3-sulfamoylindolin-2-one **3f** as major product and 1,3-dihydrobenzo[*c*]isothiazole-3-carboxamide 2,2-dioxide **2f** as a minor product.

The intramolecular competitive reactions of 2-diazo-*N*-phenyl-2-(*N*-phenylsulfamoyl)acetamides reveal that the chemoselectivity of the aromatic 1,5-C-H insertion is controlled by the electron density of the *N*-phenyl groups on amides and sulfonamides, but impacted by the steric hindrance. The results also support our proposed reaction mechanism. That is, the electrophilic attack of Cu-carbon ylides on more electron-rich phenyl group in reactants is the key step, i.e. rate-limiting step, in the aromatic carbene 1,5-C-H insertion. This result is similar to Maguire’s report that the aliphatic C-H insertion of carbenes derived from 2-diazo-2-sulfonylacetamides exclusively occurred on the *N*-alkyl groups on amides, rather than on the alkyl groups of alkanesulfonyls [31]. These results together reveal that diazosulfonamides have more inert reactivity than their analogs diazoacetamides

## 3. Materials and Methods

### 3.1. Materials and Instruments

Toluene was refluxed over Na with diphenyl ketone as an indicator and freshly distilled prior to use. Melting points were obtained on a MP-500 melting point apparatus and are uncorrected. ^1^H and ^13^C-NMR spectra were recorded on a 400 MHz Bruker spectrometer in CDCl_3_ with TMS as an internal standard and the chemical shifts (*δ*) are reported in parts per million (ppm). The IR spectra (KBr pellets, *v* (cm^−1^)) were taken on a FTIR spectrometer. HRMS measurements were carried out on an Agilent LC/MSD TOF mass spectrometer. TLC separations were performed on silica gel GF254 plates, and the plates were visualized under UV light. Petroleum ether (PE, 30−60 °C) and ethyl acetate (EA) were used for column separation.

It is noteworthy that the signal of the carbon attached with diazo group in diazosulfamoylacetamides **1** cannot be displayed in their ^13^C-NMR spectra. Similar observations were also reported by Novikov and Du Bios in the synthesis of diazosulfones and diazosulfonates, respectively [27,28].

### 3.2. General Procedure for the Preparation of Chloroacetamides ***5***

Dimethylammonium chloride (4.08 g, 50 mmol) and triethylamine (10.1 g, 100 mmol) were dissolved in dichloromethane (50 mL). Chloroacetyl chloride (6.78 g, 60 mmol) was added dropwise under stirring at 0 °C. The resulting solution was stirred at 0 °C for 4 h. After washing with water (30 mL × 3), drying over Na_2_SO_4_, and removing the solvent, the crude *N,N*-dimethyl-2-chloroacetamide was obtained and used directly in the next step [34].

To a solution of diphenylamine (8.45 g, 50 mmol) or *N*-methylaniline (5.35 g, 50 mmol) in dry toluene (30 mL), chloroacetyl chloride (5.65 g, 50 mmol) was added dropwise under stirring. The resulting solution was refluxed for 4 h. After removal of solvent under reduced pressure, the crude product was obtained and used directly in the next step [34].

#### 3.2.1. 2-Chloro-N,N-dimethylacetamide (**5a**)

Yellow oil, 6.10 g, 90% yield. ^1^H-NMR (400 MHz, CDCl_3_) δ 4.07 (s,2H), 3.08(s, 3H), 2.97(s, 3H). ^13^C-NMR (101 MHz, CDCl_3_) δ 166.5, 41.2, 37.6, 35.9 [35].

#### 3.2.2. 2-Chloro-*N*-methyl-*N*-phenylacetamide (**5c**)

Blue crystals, m.p. 76–77 °C, Lit [34]. 77–79 °C. 8.30 g, 90% yield. ^1^H-NMR (400 MHz, CDCl_3_) δ 7.46–7.29(m, 5H, ArH), 3.86 (s, 2H),3.32 (s, 3H). ^13^C-NMR (101 MHz, CDCl_3_) δ 166.2, 142.6, 130.0, 128.5, 127.0, 41.4, 37.9.

#### 3.2.3. 2-Chloro-N,N-diphenylacetamide (**5d**)

Colorless crystals, m.p. 124–125 °C, Lit [35]. 119–120 °C. 11.75 g, 96% yield. ^1^H-NMR (400 MHz, CDCl_3_) δ 7.39–7.25(m, 10H, ArH), 4.02 (s, 2H).

### 3.3. General Procedure for the Synthesis of Sulfamoylacetamides ***7***

Chloroacetamide **5** (25 mmol) was added to a suspension of sodium sulfite (3.94 g, 31.25 mmol) in water (40 mL), and the mixture was stirred for 8 h at room temperature. After removal of water under reduced pressure, the crude product containing sodium chloride was obtained. To the crude product 25 mL of toluene was added, which was removed along with water under reduced pressure. The procedure was repeated three times to give the crude dry sodium sulfonate.

To the crude dry sodium sulfonate, 12.5 mL (ca. 20.5 g, 13.5 mmol) of POCl_3_ was added and the mixture was refluxed for 3 h. After being dissolved in DCM (13 mL), to the mixture was added a secondary amine **4** (25 mmol) dissolved in DCM (13 mL). The resulting solution was stirred at room temperature for another 3 h. The mixture was transferred to conical flask of 250 mL, and 50 mL of water and ice mixture was added. After the pH value of the mixture was adjusted to 6–8 with 20% sodium hydroxide, the organic phase was separated and then dried over anhydrous sodium sulfate. After removal of solvent under reduced pressure, the residual oil was purified on silica gel column (EA:PE = 1:10, *v/v*) to yield the pure 2-sulfamoylacetamide **7**.

#### 3.3.1. 2-(*N*-Benzyl-*N*-butylsulfamoyl)-*N*-methyl-*N*-phenylacetamide (**7c**)

Yellow oil, 5.56 g, 59% yield. R*_f_* = 0.20 (silica gel plate, EA:PE = 1:5, *v/v*). ^1^H-NMR (400 MHz, CDCl_3_) δ 7.49–7.23 (m, 10H), 4.46 (s, 2H), 3.84 (s, 2H), 3.33 (s, 3H), 3.24 (t, *J* = 7.6 Hz, 2H), 1.46–1.33 (m, 2H), 1.24–1.13 (m, 2H), 0.78 (t, *J* = 7.3 Hz, 3H). ^13^C-NMR (101 MHz, CDCl_3_) δ 162.6, 143.0, 137.1, 130.1, 128.6, 128.5, 128.2, 127.6, 127.5, 54.4, 52.9, 48.8, 37.8, 30.4, 19.9, 13.6. IR (CH_2_Cl_2_, cm^−1^): 1662, 1339, 1148. HRMS (ESI) calcd for C_20_H_27_N_2_O_3_S^+^ [M + H^+^] *m/z*: 375.1737, found 375.1744.

#### 3.3.2. 2-(*N*-Benzyl-*N*-butylsulfamoyl)-*N*,*N*-diphenylacetamide (**7d**)

Yellow crystals, m.p. 111−112 °C. 3.02 g, 28% yield. R*_f_* = 0.17 (silica gel plate, EA:PE = 1:5, *v/v*). ^1^H-NMR (400 MHz, CDCl_3_) δ 7.47–7.22 (m, 15H), 4.51 (s, 2H), 4.03 (s, 2H), 3.27 (t, *J* = 7.6 Hz, 2H), 1.45 –1.39 (m, 2H), 1.23–1.17 (m, 2H), 0.80 (t, *J* = 7.4 Hz, 3H). ^13^C-NMR (101 MHz, CDCl_3_) δ 162.9, 142.0, 141.9, 137.1, 130.1, 129.2, 128.9, 128.7, 128.5, 128.1, 127.6, 127.0, 126.4, 55.4, 53.0, 48.9, 30.3, 19.8, 13.6. IR (CH_2_Cl_2_, cm^−1^): 1671, 1343, 1152. C_25_H_29_N_2_O_3_S^+^ [M + H^+^] *m/z*: 437.1893, found 437.1902.

#### 3.3.3. *N*-Methyl-2-(*N*-methyl-*N*-phenylsulfamoyl)-*N*-phenylacetamide (**7e**)

Colorless crystals, m.p. 86−88 °C. 6.29 g, 74% yield. R*_f_* = 0.11 (silica gel plate, EA:PE = 1:5, *v/v*). ^1^H-NMR (400 MHz, CDCl_3_) δ 7.60–7.56 (m, 2H), 7.45–7.32 (m, 5H), 7.31–7.23 (m, 3H), 3.78 (s, 2H), 3.46 (s, 3H), 3.34 (s, 3H). ^13^C-NMR (101 MHz, CDCl_3_) δ 162.5, 142.8, 141.0, 130.1, 129.3, 128.6, 127.6, 127.4, 127.4, 52.1, 40.2, 37.8. IR (CH_2_Cl_2_, cm^−1^): 1658, 1382, 1180. HRMS (ESI) calcd for C_16_H_18_N_2_NaO_3_S^+^ [M + Na^+^] *m/z*: 341.0930, found 341.0939.

#### 3.3.4. *N*-Methyl-*N*-phenyl-2-(*N*,*N*-diphenylsulfamoyl)acetamide (**7f**)

Green crystals, m.p. 130−131 °C, 2.85 g, 30% yield. R*_f_* = 0.10 (silica gel plate, EA:PE = 1:5, *v/v*). ^1^H-NMR (400 MHz, CDCl_3_) δ 7.57 (d, *J* = 7.6 Hz, 4H), 7.42–7.35 (m, 7H), 7.30 (d, *J* = 6.8 Hz, 2H), 7.26 (d, *J* = 7.2 Hz, 2H), 4.07 (s, 2H), 3.36 (s, 3H). ^13^C-NMR (101 MHz, CDCl_3_) δ 161.5, 142.7, 141.0, 130.0, 129.3, 128.6, 128.5, 127.5, 127.5, 77.3, 77.0, 76.7, 53.6, 37.9. IR (CH_2_Cl_2_, cm^−1^):1659, 1356, 1155. HRMS (ESI) calcd for C_21_H_21_N_2_O_3_S^+^ [M + H^+^] *m/z*: 381.1267, found 381.1263.

#### 3.3.5. 2-(*N*-Methyl-*N*-phenylsulfamoyl)-*N*,*N*-diphenylacetamide (**7g**)

Yellow crystals, m.p. 165−166 °C. 8.38 g, 88% yield. R*_f_* = 0.13 (silica gel plate, EA:PE = 1:5, *v*/*v*). ^1^H-NMR (400 MHz, CDCl_3_) δ 7.60 (d, *J* = 7.7 Hz, 2H), 7.47–7.17 (m, 13H), 3.96 (s, 2H), 3.48 (s, 3H). ^13^C-NMR (101 MHz, CDCl_3_) δ 162.8, 141.9, 140.9, 130.1, 129.3, 129.2, 128.6, 127.68, 127.5, 127.0, 126.4, 77.3, 77.0, 76.7, 52.9, 40.4. IR (CH_2_Cl_2_, cm^−1^): 1668, 1351, 1148. HRMS (ESI) calcd for C_21_H_21_N_2_O_3_S^+^ [M + H^+^] *m/z*: 381.1267, found 381.1263.

#### 3.3.6. *N*,*N*-Diphenyl-2-(*N*,*N*-diphenylsulfamoyl)acetamide (**7h**)

Blue crystals, m.p. 199−200 °C. 7.96 g, 72% yield. R*_f_* = 0.31 (silica gel plate, EA:PE = 1:3, *v*/*v*). ^1^H-NMR (400 MHz, CDCl_3_) δ 7.59 (d, J = 7.8 Hz, 4H, ArH), 7.40–7.17 (m, 16H, ArH), 4.21 (s, 2H). ^13^C-NMR (101 MHz, CDCl_3_) δ 161.9, 142.0, 141.9, 140.8, 130.0, 129.3, 129.2, 128.9, 128.8, 128.6, 127.7, 127.0, 126.5, 54.4. IR (CH_2_Cl_2_, cm^−1^): 1669, 1357, 1159. HRMS (ESI) calcd for C_26_H_23_N_2_O_3_S^+^ [M + H^+^] *m/z*: 443.1424, found 443.1419.

### 3.4. General Procedure for the Synthesis of 2-Diazo-2-sulfamoylacetamides ***1***

The corresponding 2-sulfamoylacetamide **7** (3 mmol) was dissolved in 24 mL of dry MeCN with 4-nitrobenzenesulfonyl azide (699 mg, 3.06 mmol). The resulting solution was cooled to 0 °C. DBU (684 mg, 0.69 mL, 4.5 mmol) was added, and the reaction mixture was warmed up to room temperature and stirred for 2 h. After removal of solvent under reduced pressure, the crude product was purified on silica gel column (EA:PE = 1:10, *v/v*) to yield the pure 2-diazo-2-sulfamoylacetamides **1**.

#### 3.4.1. 2-Diazo-*N,N*-dimethyl-2-(*N,N*-diphenylsulfamoyl)acetamide (**1b**)

Yellow crystals, m.p. 107−108 °C. R*_f_* = 0.16 (silica gel plate, EA:PE = 1:5, *v/v*). 966 mg, 88% yield. ^1^H-NMR (400 MHz, CDCl_3_) δ 7.64 (d, *J* = 7.6 Hz, 2H), 7.45–7.25 (m, 4H), 7.37–7.27 (m, 4H), 4.00 (s, 3H), 3.52 (s, 3H). ^13^C-NMR (101 MHz, CDCl_3_) δ 162.8, 141.9, 130.1, 129.3, 128.6, 127.68, 127.5, 127.0, 126.4, 52.9, 40.4. IR (CH_2_Cl_2_, cm^−1^): 2097, 1641, 1357, 1143. HRMS (ESI) calcd for C_16_H_16_N_4_NaO_3_S^+^ [M + Na^+^] *m/z*: 367.0835, found 367.0840.

#### 3.4.2. 2-(*N*-Benzyl-*N*-butylsulfamoyl)-2-diazo-*N*-methyl-*N*-phenyl acetamide (**1c**)

Yellow crystals, m.p. 108−110 °C. 540 mg, 45% yield. R*_f_* = 0.33 (silica gel plate, EA:PE = 1:5, *v/v*). ^1^H-NMR (400 MHz, CDCl_3_) δ 7.46–7.21 (m, 10H), 4.59 (s, 2H), 3.35 (s, 3H), 3.32 (t, *J* = 7.2 Hz, 2H), 1.46–1.38 (m, 2H), 1.46–1.38 (m, 2H), 1.26–1.16 (m, 2H), 0.80 (t, *J* = 7.6 Hz, 3H). ^13^C-NMR (101 MHz, CDCl_3_) δ 159.5, 142.3, 137.0, 130.3, 128.5, 128.5, 128.1, 127.6, 126.5, 53.5, 49.6, 38.3, 30.3, 19.8, 13.6. IR (CH_2_Cl_2_, cm^−1^): 2093, 1642, 1347, 1149. HRMS (ESI) calcd for C_20_H_25_N_4_O_3_S^+^ [M + H^+^] *m/z*: 401.1642, found 401.1654.

#### 3.4.3. 2-(*N*-Benzyl-*N*-butylsulfamoyl)-2-diazo-*N,N*-diphenyl acetamide (**1d**)

Yellow oil, R*_f_* = 0.58 (silica gel plate, EA:PE = 1:5, *v/v*). 693 mg, 50% yield. ^1^H-NMR (400 MHz, CDCl_3_) δ 7.45–7.24 (m, 15H), 4.62 (s, 2H), 3.41 (d, *J* = 7.6 Hz, 2H), 1.54–1.46 (m, 2H), 1.32–1.23 (m, 2H), 0.86 (t, *J* = 7.6 Hz, 3H). ^13^C-NMR (101 MHz, CDCl_3_) δ C (101 MHz, CDCl_3_) 160.2, 141.9, 136.9, 129.8, 128.5, 128.1, 127.7, 127.5, 126.8, 53.4, 49.6, 30.3, 19.9, 13.7. IR (CH_2_Cl_2_, cm^−1^): 2103, 1612, 1343, 1148. HRMS (ESI) calcd for C_25_H_27_N_4_O_3_S^+^ [M + H^+^] *m/z*: 463.1798, found 463.1796.

#### 3.4.4. 2-Diazo-*N*-methyl-2-(*N*-methyl-*N*-phenylsulfamoyl)-*N*-phenylacetamide (**1e**)

Yellow crystals, m.p. 107−108 °C. 966 mg, 88% yield. R*_f_* = 0.16 (silica gel plate, EA:PE = 1:5, *v/v*). ^1^H-NMR (400 MHz, CDCl_3_) δ 7.50–7.44 (m, 2H), 7.43–7.36 (m, 2H), 7.37–7.27 (m, 4H), 7.08–7.02 (m, 2H), 3.60 (s, 3H), 3.34 (s, 3H). ^13^C-NMR (101 MHz, CDCl_3_) δ 159.4, 142.2, 140.8, 130.2, 129.3, 128.3, 127.6, 126.3, 126.3, 40.4, 38.3. IR (CH_2_Cl_2_, cm^−1^): 2097, 1641, 1357, 1143. HRMS (ESI) calcd for C_16_H_16_N_4_NaO_3_S^+^ [M + Na^+^] *m*/*z*: 367.0835, found 367.0840.

#### 3.4.5. 2-Diazo-*N*-methyl-*N*-phenyl-2-(*N,N*-diphenylsulfamoyl)acetamide (**1f**)

Yellow crystals, m.p. 127−128 °C. 316 mg, 26% yield. R*_f_* = 0.30 (silica gel plate, EA:PE = 1:5, *v/v*). ^1^H-NMR (400 MHz, CDCl_3_) δ 7.41 (d, *J* = 7.2 Hz, 4H), 7.38–7.29 (m, 9H), 6.89–6.86 (m, 2H), 3.33 (s, 3H). ^13^C-NMR (101 MHz, CDCl_3_) δ 158.3, 142.2, 140.9, 130.3, 129.4, 128.4, 128.2, 127.5, 126.2, 38.4. IR (CH_2_Cl_2_, cm^−1^): 2102, 1642, 1355, 1162. HRMS (ESI) calcd for C_21_H_19_N_4_O_3_S^+^ [M + H^+^] *m/z*: 407.1172, found 407.1165.

#### 3.4.6. 2-Diazo-2-(*N*-methyl-*N*-phenylsulfamoyl)-*N,N*-diphenylacetamide (**1g**)

Yellow crystals, m.p. 127−128 °C. 731 mg, 60% yield. R*_f_* = 0.26 (silica gel plate, EA:PE = 1:5, *v/v*). ^1^H-NMR (400 MHz, CDCl_3_) δ 7.51 (d, *J* = 7.8 Hz, 2H), 7.45 (t, *J* = 7.7 Hz, 2H), 7.40–7.29 (m, 5H), 7.25 (d, *J* = 7.5 Hz, 2H), 7.06 (d, *J* = 7.5 Hz, 4H), 3.57 (s, 3H). ^13^C-NMR (101 MHz, CDCl_3_) δ 160.0, 141.7, 140.8, 129.8, 129.4, 127.6, 127.4, 126.7, 126.1, 40.4. IR (CH_2_Cl_2_, cm^−1^): 2099, 1650, 1354, 1144. HRMS (ESI) calcd for C_21_H_19_N_4_O_3_S^+^ [M + H^+^] *m/z*: 407.1172, found 407.1174.

#### 3.4.7. 2-Diazo-*N,N*-diphenyl-2-(*N,N*-diphenylsulfamoyl)acetamide (**1h**)

Yellow crystals, m.p. 178−180 °C. 913 mg, 65% yield. R*_f_* = 0.60 (silica gel plate, EA:PE = 1:3, *v/v*). ^1^H-NMR (400 MHz, CDCl_3_) δ 7.64 (d, *J* = 8.0 Hz, 4H, ArH), 7.39 (t, *J* = 7.6 Hz, 4H, ArH), 7.35–7.20 (m, 8H, ArH), 6.93 (d, *J* = 7.6 Hz, 4H, ArH). ^13^C-NMR (101 MHz, CDCl_3_) δ 158.7, 141.8, 140.7, 129.8, 129.4, 128.4, 127.6, 127.3, 126.6. IR (CH_2_Cl_2_, cm^−1^): 2104, 1656, 1350, 1155. HRMS (ESI) calcd for C_26_H_21_N_4_O_3_S^+^ [M + H^+^] *m*/*z*: 469.1329, found 469.1329.

### 3.5. General Procedure for the Reaction of 2-Diazo-2-sulfamoylacetamides ***1***

To a suspension of Cu(acac)_2_ (2 mol%, 13 mg, 0.01 mmol) in 10 mL of toluene, the corresponding diazosulfamoylacetamide **1** (0.5 mmol) was added, and the mixture was refluxed for 9 h. After removal of solvent under reduced pressure, the crude reaction mixture was purified on silica gel column (PE:EA = 10:1, *v/v*) to afford the desired product **2** and/or **3**.

#### 3.5.1. *N,N*-Dimethyl-2,2-dioxido-1-phenyl-1,3-dihydrobenzo[c]isothiazole-3-carboxamide (**2b**)

Colorless crystals, 142 mg, 90% yield, m.p. 86−88 °C, R*_f_* = 0.10 (silica gel plate, EA:PE = 1:5, *v/v*). ^1^H-NMR (400 MHz, CDCl_3_) δ: 7.54–7.40 (m, 5H), 7.34–7.21 (m, 2H), 7.09 (td, *J* = 7.6, 1.1 Hz, 1H), 6.65 (d, *J* = 8.0 Hz, 1H), 5.53 (s, 1H), 3.34 (s, 3H), 3.10 (s, 3H). ^13^C-NMR (101 MHz, CDCl_3_) δ: 161.8, 141.8, 133.6, 130.0, 129.1, 128.4, 126.8, 123.0, 119.8, 111.9, 63.2, 37.9, 36.7. IR (KBr, cm^−1^): 1656, 1332, 1160. HRMS (ESI) calcd for C_16_H_17_N_2_O_3_S^+^ [M + H^+^] *m/z*: 317.0954, found 317.0953.

#### 3.5.2. *N*-Benzyl-*N*-butyl-2-oxo-1-methylindoline-3-sulfonamide (**3c**)

Yellow oil, 142 mg, 76% yield, R*_f_* = 0.18 (silica gel plate, EA:PE = 1:5, *v/v*). ^1^H-NMR (400 MHz, CDCl_3_) δ 7.65 (d, *J* = 7.5 Hz, 1H), 7.41–7.27 (m, 6H), 7.13 (t, *J* = 7.6 Hz, 1H), 6.86 (d, *J* = 7.9 Hz, 1H), 4.84 (s, 1H), 4.63 (d, *J* = 15.6 Hz, 1H), 4.39 (d, *J* = 15.6 Hz, 1H), 3.47–3.34 (m, 1H), 3.23 (s, 3H), 3.20–3.10 (m, 1H), 1.44–1.32 (m, 2H), 1.19–1.09 (m, 2H), 0.79 (t, *J* = 7.3 Hz, 3H). ^13^C-NMR (101 MHz, CDCl_3_) δ 167.7, 144.8, 138.3, 136.6, 130.2, 128.5, 128.2, 127.7, 126.7, 123.2, 119.6, 108.5, 65.5, 52.8, 48.7, 30.3, 26.6, 19.8, 13.6. IR (CH_2_Cl_2_, cm^−1^): 1721, 1372, 1343, 1148. HRMS (ESI) calcd for C_20_H_25_N_2_O_3_S^+^ [M + H^+^] *m/z*: 373.1580, found 373.1584.

#### 3.5.3. *N*-Benzyl-*N*-butyl-2-oxo-1-phenylindoline-3-sulfonamide (**3d**)

Yellow oil, R*_f_* = 0.18 (silica gel plate, EA:PE = 1:5, *v/v*). 184 mg, 85% yield. ^1^H-NMR (400 MHz, CDCl_3_) δ 7.74 (d, *J* = 7.5 Hz, 1H), 7.57 (t, *J* = 7.6 Hz, 2H), 7.51–7.39 (m, 5H), 7.39–7.27 (m, 4H), 7.20 (t, *J* = 7.6 Hz, 1H), 6.84 (d, *J* = 7.9 Hz, 1H), 5.02 (s, 1H), 4.74 (d, *J* = 15.6 Hz, 1H), 4.46 (d, *J* = 15.6 Hz, 1H), 3.54–3.44 (m, 1H), 3.30–3.19 (m, 1H), 1.57–1.39 (m, 2H), 1.34–1.13 (m, 2H), 0.82 (t, *J* = 7.3 Hz, 3H). ^13^C-NMR (101 MHz, CDCl_3_) δ 167.4, 145.0, 136.6, 133.7, 130.1, 129.8, 128.6, 128.6, 128.2, 127.7, 127.0, 126.6, 123.7, 119.5, 109.8, 65.7, 53.1, 48.8, 30.3, 19.8, 13.6. IR (CH_2_Cl_2_, cm^−1^): 1724, 1372, 1346, 1148. HRMS (ESI) calcd for C_25_H_27_N_2_O_3_S^+^ [M + H^+^] *m/z*: 435.1737, found 435.1740.

#### 3.5.4. *N*,1-Dimethyl-2-oxo-*N*-phenylindoline-3-sulfonamide (**3e**)

Colorless crystals, m.p. 127−128 °C. 84 mg, 53% yield. R*_f_* = 0.14 (silica gel plate, EA:PE = 1:5, *v/v*). ^1^H-NMR (400 MHz, CDCl_3_) δ 7.69–7.63 (m, 2H), 7.50 (d, *J* = 7.5 Hz, 1H), 7.42 (t, *J* = 7.7 Hz, 2H), 7.38–7.31 (m, 2H), 7.07 (t, *J* = 7.6 Hz, 1H), 6.84 (d, *J* = 7.8 Hz, 1H), 4.88 (s, 1H), 3.52 (s, 3H), 3.25 (s, 3H). ^13^C-NMR (101 MHz, CDCl_3_) δ 167.8, 144.9, 140.8, 130.2, 129.5, 128.0, 127.9, 126.7, 123.3, 119.1, 108.5, 63.3, 41.5, 26.6. IR (CH_2_Cl_2_, cm^−1^): 1720, 1372, 1348, 1146. HRMS (ESI) calcd for C_16_H_17_N_2_O_3_S^+^ [M + H^+^] *m*/*z*: 317.0954, found 317.0959.

The reaction of 2-diazo-2-sulfamoylacetamide **1a** gave rise to inseparable isomeric products **2f** and **3f** with very similar polarity under the same reaction conditions. Their yields were obtained on the basis of 1H NMR analysis. Their melting point is the melting point of the isomeric mixture.

#### 3.5.5. *N*-Methyl-2,2-dioxido-*N*,1-diphenyl-1,3-dihydrobenzo[c]isothiazole-3-carboxamide (**2f**)

Colorless crystals, 28 mg, 15% yield, m.p. 115−117 °C, R*_f_* = 0.18 (silica gel plate, EA:PE = 1:5, *v/v*). ^1^H-NMR (400 MHz, CDCl_3_) δ: 7.63–7.62 (m, 2H), 7.50–7.48 (m, 4H), 7.34–7.21 (m, 2H), 7.09 (td, *J* = 7.6 Hz, 2H), 7.01 (d, *J* = 7.6 Hz, 1H), 6.82 (d, *J* = 7.6 Hz, 3H), 6.72 (d, *J* = 8.0 Hz, 1H), 4.72 (s, 1H), 3.05 (s, 3H). HRMS (ESI) calcd for C_21_H_19_N_2_O_3_S^+^ [M + H^+^] *m/z*: 379.1111, found 379.1107.

#### 3.5.6. 1-Methyl-2-oxo-*N,N*-diphenylindoline-3-sulfonamide (**3f**)

Colorless crystals, 83 mg, 44% yield, m.p. 115−117 °C. R*_f_* = 0.18 (silica gel plate, EA:PE = 1:5, *v/v*). ^1^H-NMR (400 MHz, CDCl_3_) δ 7.58 (d, *J* = 7.6 Hz, 1H), 7.52 – 7.46 (m, 1H), 7.43–7.21 (m, 10H), 7.09 (t, *J* = 7.6 Hz, 1H), 6.83 (d, *J* = 7.8 Hz, 1H), 5.05 (s, 1H), 3.21 (s, 3H). ^13^C-NMR (101 MHz, CDCl_3_) δ 166.7, 144.9, 140.9, 130.4, 129.8, 129.3, 129.2, 129.1, 128.4, 127.8, 127.1, 123.2, 123.1, 118.9, 108.5, 63.6, 26.7. IR (CH_2_Cl_2_, cm^−1^): 1722, 1369, 1144. HRMS (ESI) calcd for C_21_H_19_N_2_O_3_S^+^ [M + H^+^] *m/z*: 379.1111, found 379.1107.

#### 3.5.7. *N*-Methyl-2-oxo-*N*,1-diphenylindoline-3-sulfonamide (**3g**)

Colorless crystals, m.p. 127−129 °C. 98 mg, 52% yield. R*_f_* = 0.26 (silica gel plate, EA:PE = 1:5, *v/v*). ^1^H-NMR (400 MHz, CDCl_3_) δ 7.71 (d, *J* = 8.0 Hz, 2H), 7.61–7.51 (m, 3H), 7.50–7.40 (m, 5H), 7.40–7.32 (m, 1H), 7.32–7.22 (m, 1H), 7.10 (t, *J* = 7.6 Hz, 1H), 6.79 (d, *J* = 7.9 Hz, 1H), 5.03 (s, 1H), 3.55 (s, 3H). ^13^C NMR (101 MHz, CDCl_3_) δ 167.5, 145.2, 140.8, 133.7, 130.1, 129.8, 129.6, 128.7, 128.1, 128.1, 127.0, 126.6, 123.7, 119.0, 109.8, 63.5, 41.7. IR (CH_2_Cl_2_, cm^−1^): 1724, 1348, 1146. HRMS (ESI) calcd for C_21_H_19_N_2_O_3_S^+^ [M + H^+^] *m/z*: 379.1111, found 379.1107.

#### 3.5.8. 2-Oxo-*N*,*N*,1-triphenylindoline-3-sulfonamide (**3h**)

Colorless crystals, m.p. 68–70 °C. 106 mg, 48% yield. R*_f_* = 0.24 (silica gel plate, EA:PE = 1:5, *v/v*). ^1^H-NMR (400 MHz, CDCl_3_) δ 7.81 (d, *J* = 7.5, 1H), 7.51–7.40 (m, 4H), 7.40–7.27 (m, 4H), 7.21–7.04 (m, 6H), 6.84 (d, *J* = 8.0 Hz, 2H), 6.62 (d, *J* = 8.0 Hz, 1H), 6.26 (s, 1H), 5.03 (s, 1H). ^13^C-NMR (101 MHz, CDCl_3_) δ 166.5, 149.7, 145.0, 139.7, 133.6, 131.7, 130.4, 129.7, 129.6, 128.6, 127.5, 126.4, 124.8, 124.2, 123.5, 121.4, 118.7, 113.8, 109.6, 69.0. IR (CH_2_Cl_2_, cm^−1^): 1722, 1372, 1369, 1140. HRMS (ESI) calcd for C_26_H_21_N_2_O_3_S^+^ [M + H^+^] *m/z*: 441.1267, found 441.1266.

^1^H-NMR and ^13^C-NMR spectra of unknown compounds **1**, **2**, **3**, and **7** are included in The Supplementary Materials.

## 4. Conclusions

The intramolecular C-H insertions of carbenes derived from different diazosulfamoylacetamides were studied. The results reveal that the aromatic 1,5-C-H insertion can occur on the *N*-phenylsulfonamides only when they have no *N*-phenyl group on their acetamide. No aliphatic C-H insertions were observed for diazo(*N,N*-dialkylsulfamoyl)acetamides. The intramolecular competitive aromatic 1,5-C-H insertion of *N*-aryl-2-(*N*-arylsulfamoyl)-2-diazoacetamides occurs on the *N*-phenylacetamides predominantly rather than the phenyl group of *N*-phenylsulfonamides. The chemoselectivity is controlled by the electron density of the phenyl group and affected by the steric hindrance. The aromatic 1,5-C-H insertion occurs on the more electron-rich *N*-phenyl group because the key step (i.e, rate-determining step) is electrophilic attack (Frediel–Craft alkylation).

## Figures and Tables

**Table 1:** Synthesis of 2-diazo-2-sulfamoylacetamides **1.**

Entry	R^1^	R^2^	R^3^	R^4^	Yield (%)		
					5	7	1
**a**	Me	Me	Me	Ph	90	-	-
**b**	Me	Me	Ph	Ph		25	16
**c**	Me	Ph	Bu	Bn	90	59	45
**d**	Ph	Ph	Bu	Bn	96	28	50
**e**	Me	Ph	Me	Ph	90	74	88
**f**	Me	Ph	Ph	Ph		30	26
**g**	Ph	Ph	Me	Ph	96	88	60
**h**	Ph	Ph	Ph	Ph		72	65

**Table 2:** Intramolecular carbene C-H insertions of diazosulfamoylacetamides **1** ^a^.

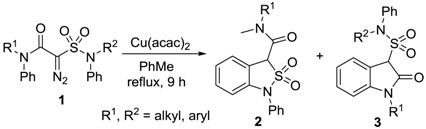
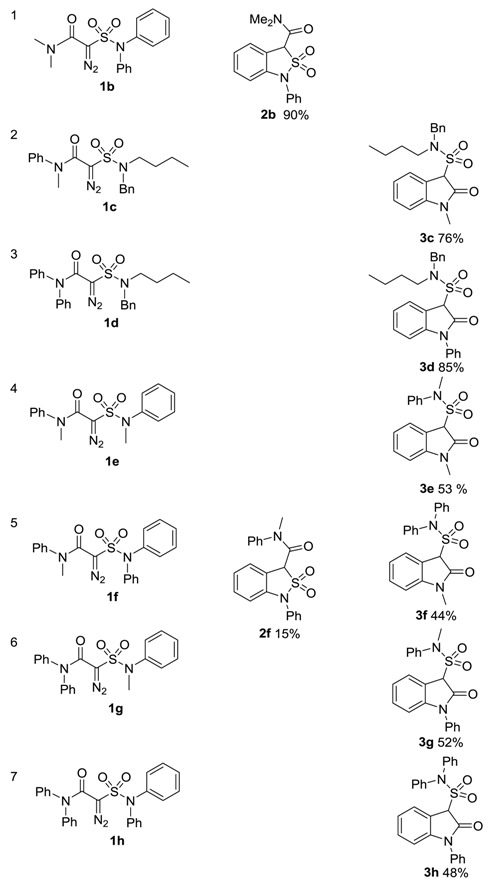

^a^ Reactions were conducted on a 0.5 mmol scale in 10 mL of toluene. The yields were obtained by chromatography on silica gel.

## Supplementary Materials

Copies of ^1^H-NMR and ^13^C-NMR spectra of unknown compounds **1**, **2**, **3**, and **7** are included in the Supporting Information

## References

[1] Padwa A., Krunpe K.E. (1992). Application of Intramolecular Carbenoid Reactions in Organic Synthesis. Tetrahedron.

[2] Doyle M.P., McKervey M.A., Ye T. (2008). Modern Catalytic Methods for Organic Synthesis with Diazo Compounds.

[3] Doyle M.P., Duffy R., Ratnikov M., Zhou L. (2010). Catalytic Carbene Insertion into C−H Bonds. Chem. Rev..

[4] Allen S.E., Walvoord R.R., Padilla-Salinas R., Kozlowski M.C. (2013). Aerobic Copper-Catalyzed Organic Reactions. Chem. Rev..

[5] Doyle M.P., Hu W., Wee A.G.H., Wang Z., Duncan S.C. (2003). Influences of Catalyst Configuration and Catalyst Loading on Selectivities in Reactions of Diazoacetamides. Barrier to Equilibrium between Diastereomeric Conformations. Org. Lett..

[6] Yoon C.H., Niagle A., Chen C., Gandhi D., Jung K.W. (2003). γ-Lactam Synthesis via C−H Insertion:  Elaboration of *N*-Benzyl Protecting Groups for High Regioselectivity toward the Total Synthesis of Rolipram. Org. Lett..

[7] Choi M.K.W., Yu W.Y., Che C.M. (2005). Ruthenium-Catalyzed Stereoselective Intramolecular Carbenoid C−H Insertion for β- and γ-Lactam Formations by Decomposition of α-Diazoacetamides. Org. Lett..

[8] Candeias N.R., Gois P.M.P., Afonso C.A.M. (2006). Rh(II)-Catalyzed Intramolecular C−H Insertion of Diazo Substrates in Water:  Scope and Limitations. J. Org. Chem..

[9] Bachmann D.G., Schmidt P.J., Geigle S.N., Chougnet A., Woggon W.-D., Gillingham D.G. (2015). Modular Ligands for Dirhodium Complexes Facilitate Catalyst Customization. Adv. Synth. Catal..

[10] Etkin N., Babu S.D., Fooks C.J., Durst T. (1990). Preparation of 3-acetyl-2-hydroxyindoles via rhodium carbenoid aromatic carbon-hydrogen insertion. J. Org. Chem..

[11] Wee A.G.H., Liu B., Zhang L. (1992). Dirhodium tetraacetate catalyzed carbon-hydrogen insertion reaction in N-substituted .alpha.-carbomethoxy-.alpha.-diazoacetanilides and structural analogs. Substituent and conformational effects. J. Org. Chem..

[12] Wang H.L., Li Z., Wang G.W., Yang S.D. (2011). Silver catalyzed intramolecular cyclization for synthesis of 3-alkylideneoxindoles via C–H functionalization. Chem. Commun..

[13] Brown D.S., Elliott M.C., Moody C.J., Mowlem T.J., Marino J.P., Padwa A. (1994). Ligand Effects in the Rhodium(II)-Catalyzed Reactions of .alpha.-Diazoamides. Oxindole Formation is Promoted by the Use of Rhodium(II) Perfluorocarboxamide Catalysts. J. Org. Chem..

[14] Mo S.Y., Yang Z.H., Xu J.X. (2014). Aqueous copper nitrate catalyzed synthesis of 3-alkylideneoxindoles from α-diazo-β-ketoanilides. Eur. J. Org. Chem..

[15] Mo S.Y., Tu J.Z., Xu J.X. (2016). Chemospecific synthesis of 2-acetoxyindole-3-carbonitriles catalyzed by Cu(acac)_2_. Russ. Chem. Bull..

[16] Merlic C.A., Zechman A.L., Miller M.M. (2001). Reactivity of (η^6^-Arene)tricarbonylchromium Complexes with Carbenoids:  Arene Activation or Protection?. J. Am. Chem. Soc..

[17] Park C.P., Nagle A., Yoon C.H., Chen C.L., Jung K.W. (2009). Formal Aromatic C−H Insertion for Stereoselective Isoquinolinone Synthesis and Studies on Mechanistic Insights into the C−C Bond Formation. J. Org. Chem..

[18] Wang J.X., Stefane B., Jaber D., Smith J.A.I., Vickery C., Diop M., Sintim H.O. (2010). Remote C-H Functionalization: Using the N-O Moiety as an Atom-Economical Tether to Obtain 1,5-and the Rare 1,7-C-H Insertions. Angew. Chem. Int. Ed..

[19] Lo V.K.Y., Guo Z., Choi M.K.W., Yu W.Y., Huang J.S., Che C.-M. (2012). Highly Selective Intramolecular Carbene Insertion into Primary C–H Bond of α-Diazoacetamides Mediated by a (*p*-Cymene) ruthenium(II) Carboxylate Complex. J. Am. Chem. Soc..

[20] Mo S.Y., Xu J.X. (2014). Chemospecific intramolecular Büchner reaction catalyzed by copper(II) acetylacetonate. ChemCatChem.

[21] Mo S.Y., Li X.H., Xu J.X. (2014). In situ generated iodonium ylides as safe carbene precursors for the chemoselective intramolecular Buchner reaction. J. Org. Chem..

[22] Dai M., Danishefsky S.J. (2007). The Total Synthesis of Spirotenuipesines A and B. J. Am. Chem. Soc..

[23] Zhang Y., Danishefsky S.J. (2010). Total Synthesis of (±)-Aplykurodinone-1: Traceless Stereochemical Guidance. J. Am. Chem. Soc..

[24] Yang Z.H., Xu J.X. (2014). Synthesis of benzo-γ-sultams via the Rh-catalyzed aromatic C-H functionalization of diazosulfonamides. Chem. Commun..

[25] Huang P.P., Yang Z.H., Xu J.X. (2017). Specific intramolecular aromatic C−H insertion of diazosulfonamides. Tetrahedron.

[26] Hrytsak M., Etkin N., Durst T. (1986). Intramolecular rhodium carbenoid insertions into aromatic C-H bonds. Preparation of 1-carboalkoxy-1,3-dihydrobenzo[*c*]thiophene 2,2-dioxides. Tetrahedron Lett..

[27] John J.P., Novikov A.V. (2007). Selective Formation of Six-Membered Cyclic Sulfones and Sulfonates by C−H Insertion. Org. Lett..

[28] Wolckenhauer S.A., Devlin A.S., Du Bois J. (2007). δ-Sultone Formation Through Rh-Catalyzed C−H Insertion. Org. Lett..

[29] Flynn C.J., Elcoate C.J., Lawrence S.E., Maguire A.R. (2010). Highly Enantioselective Intramolecular Copper Catalyzed C−H Insertion Reactions of α-Diazosulfones. J. Am. Chem. Soc..

[30] Jungon C.S., Novikov A.V. (2013). Asymmetric intramolecular C–H insertion of sulfonyldiazoacetates catalyzed by Rh(II). Tetrahedron Asymmetry.

[31] Clarke L.A., Ring A., Ford A., Sinha A.S., Lawrence S.E., Maguire A.R. (2014). Enantioselective copper catalysed C–H insertion reaction of 2-sulfonyl-2-diazoacetamides to form γ-lactams. Org. Biomol. Chem..

[32] Cui X., Xu X., Jin L.-M., Wojtas L., Zhang X.P. (2015). Stereoselective radical C–H alkylation with acceptor/acceptor-substituted diazo reagents via Co(II)-based metalloradical catalysis. Chem. Sci..

[33] Smith M.B., March J. (2004). Advanced Organic Chemistry.

[34] Pasquinucci L., Prezzavento O., Marrazzo A., Amata E., Ronsisvalle S., Georgoussi Z., Fourla D.D., Scoto G.M., Parenti C., Arico G. (2010). Evaluation of *N*-substitution in 6,7-benzomorphan compounds. Bioor Med. Chem..

[35] Hennessy E.J., Buchwald S.L. (2003). Synthesis of Substituted Oxindoles from α-Chloroacetanilides via Palladium-Catalyzed C−H Functionalization. J. Am. Chem. Soc..

