# Supplementary material for: Intramolecular Carbene C-H Insertion Reactions of 2-Diazo-2-sulfamoylacetamides
Source: Molecules. 2019 Jul 19;24(14):2628. doi: 10.3390/molecules24142628 (PMC6680402; doi:10.3390/molecules24142628)

Intramolecular carbene C-H insertion reactions of 2-diazo-2-sulfamoylacetamides

Chuqiang Que‡, Peipei Huang‡, Zhanhui Yang, Ning Chen, and Jiaxi Xu*

State Key Laboratory of Chemical Resource Engineering, Department of Organic Chemistry, College of Chemistry, BeijingUniversity of Chemical Technology, Beijing 100029, P. R. China

Supporting Information

Content

Copies of ^{1}H and ^{13}C NMR spectra of unknown compounds……………….……S2

Copies of ^{1}H and ^{13}C NMR spectra of unknown compounds

2-(*N*-Benzyl-*N*-butylsulfamoyl)-*N*,*N*-diphenyl acetamide (7c):

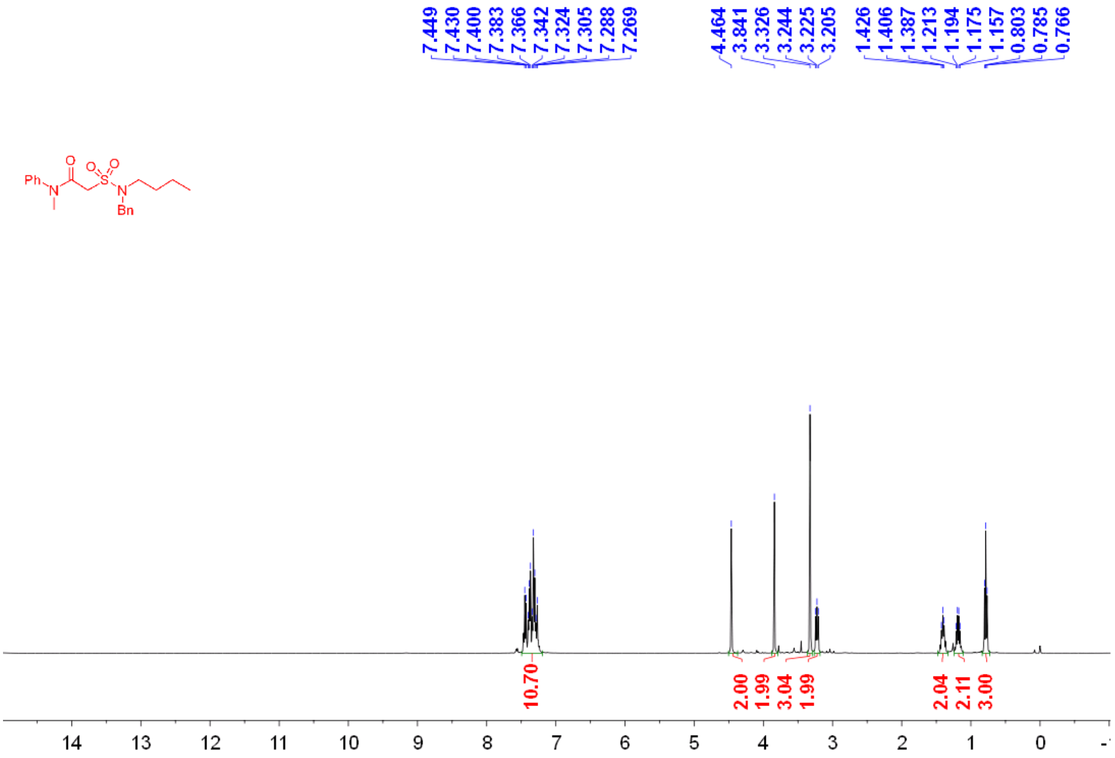

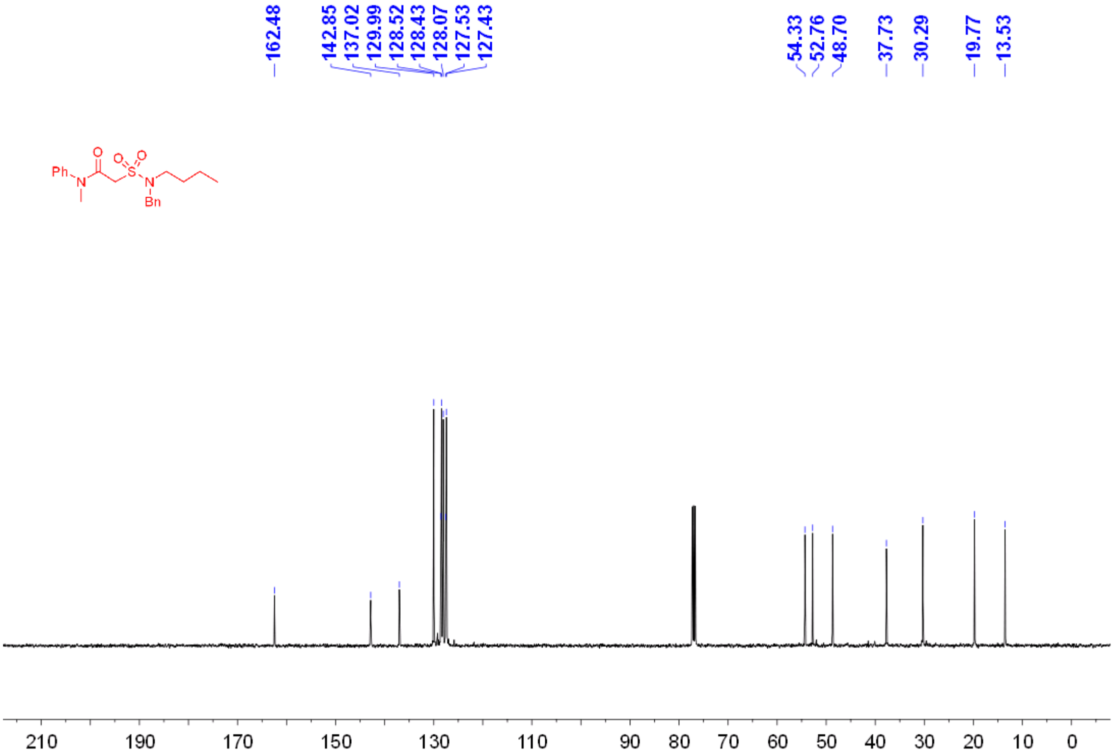

2-(*N*-Benzyl-*N*-butylsulfamoyl)-*N*,*N*-diphenyl acetamide (7d)

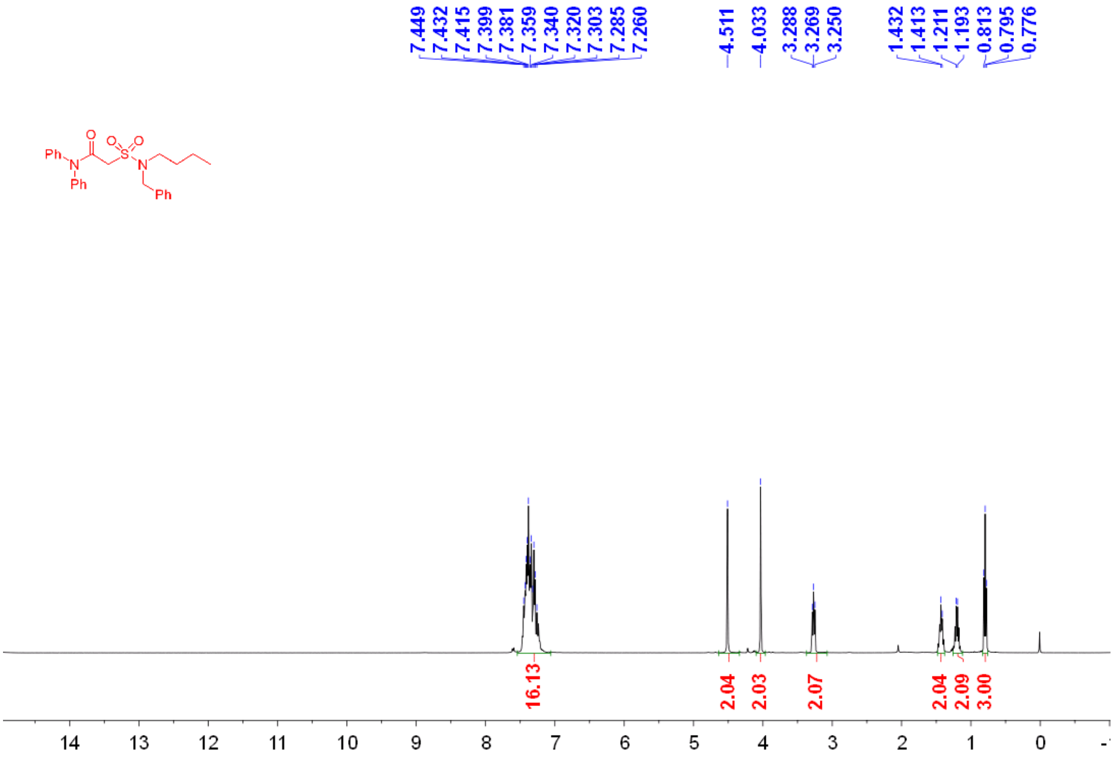

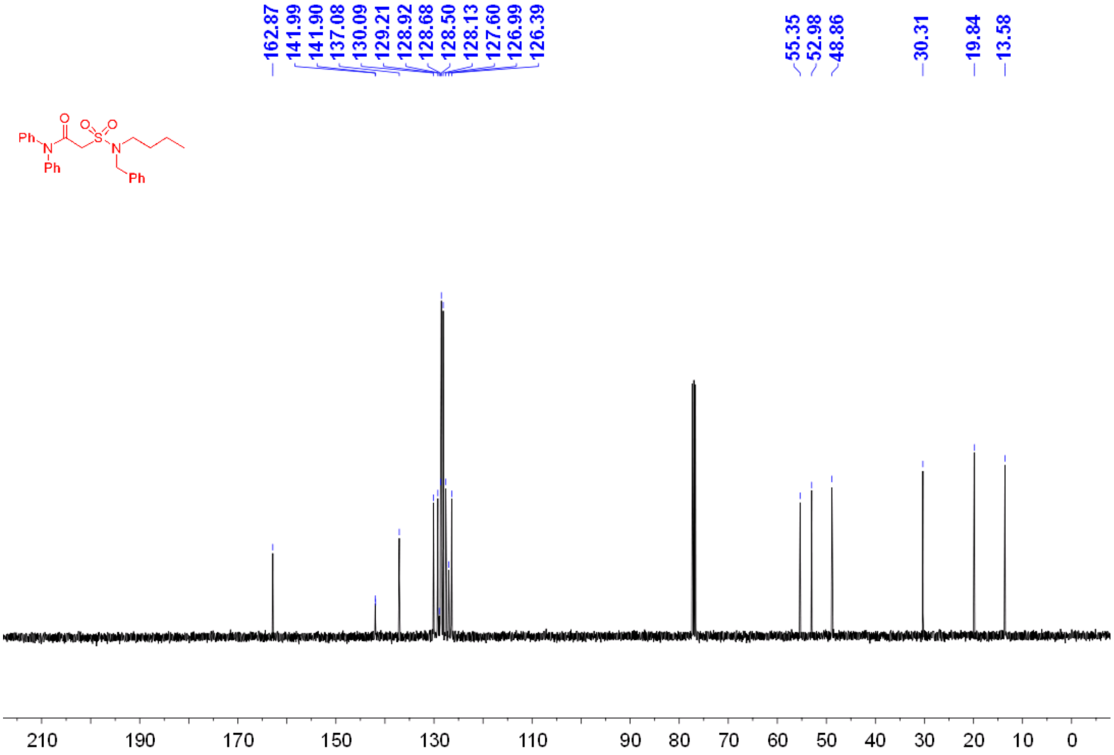

***N*-Methyl-2-(*N*-methyl-*N*-phenylsulfamoyl)-*N*-phenylacetamide (7e):**

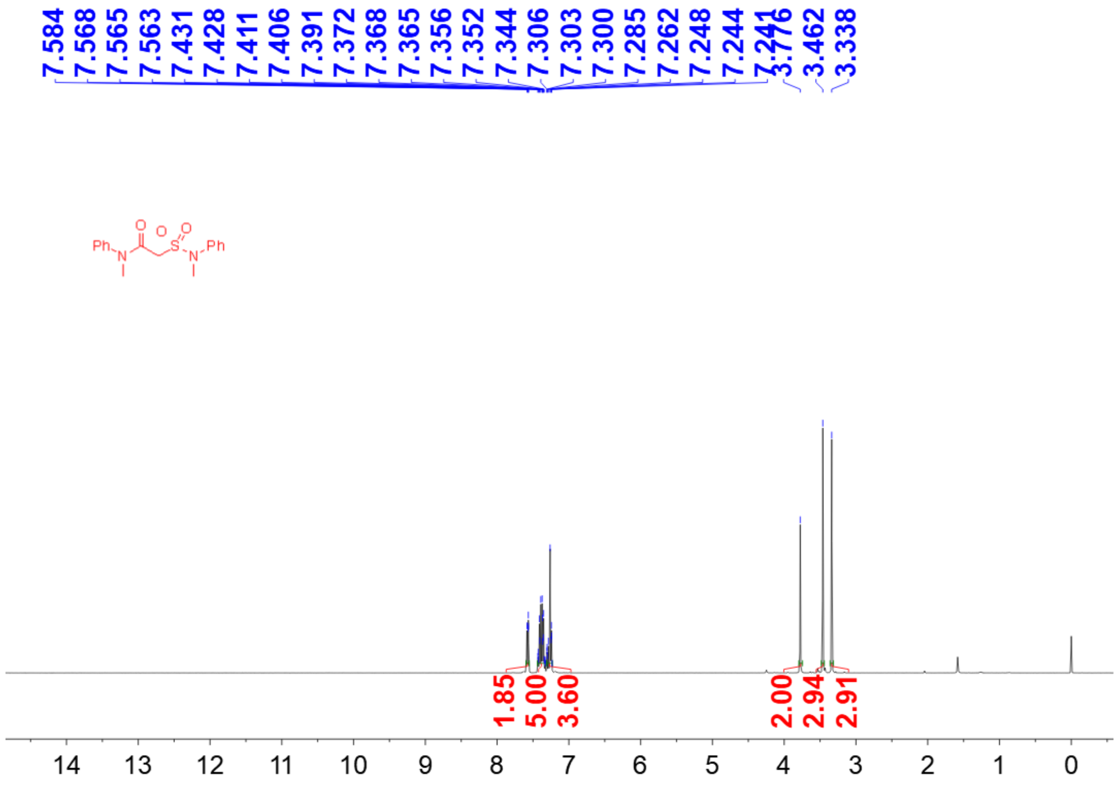

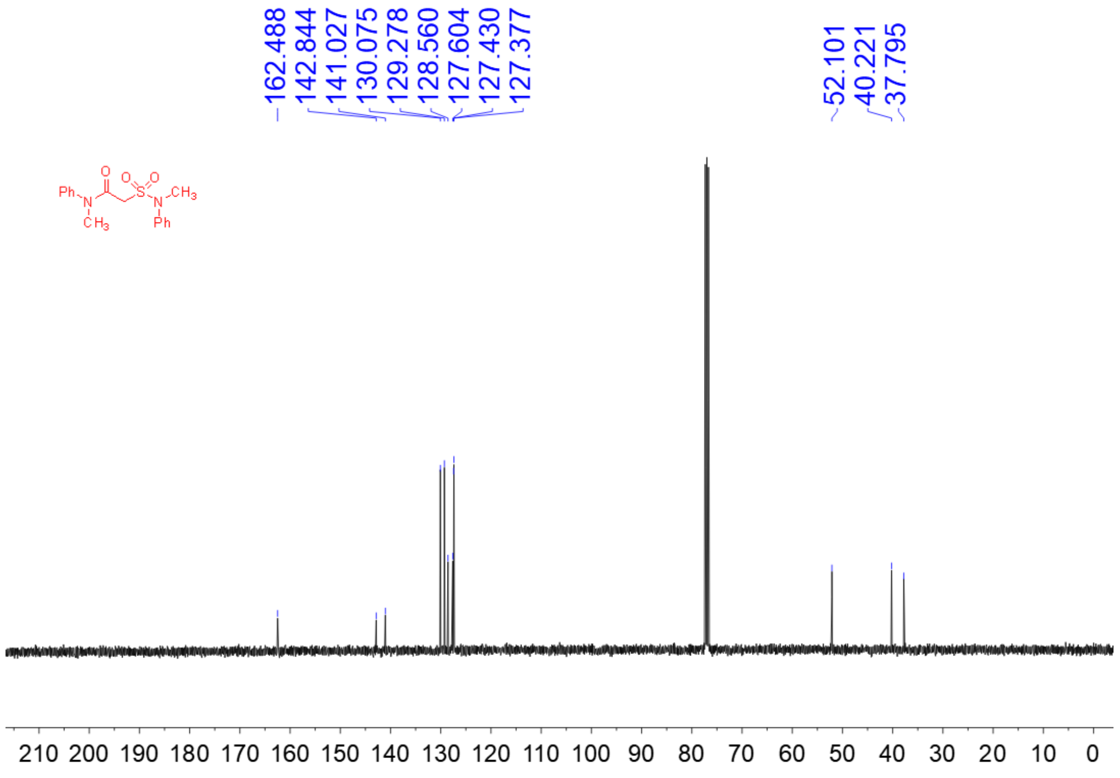

_N_-Methyl-_N_-phenyl-2-(_N_,_N_-diphenylsulfamoyl)acetamide (7f)

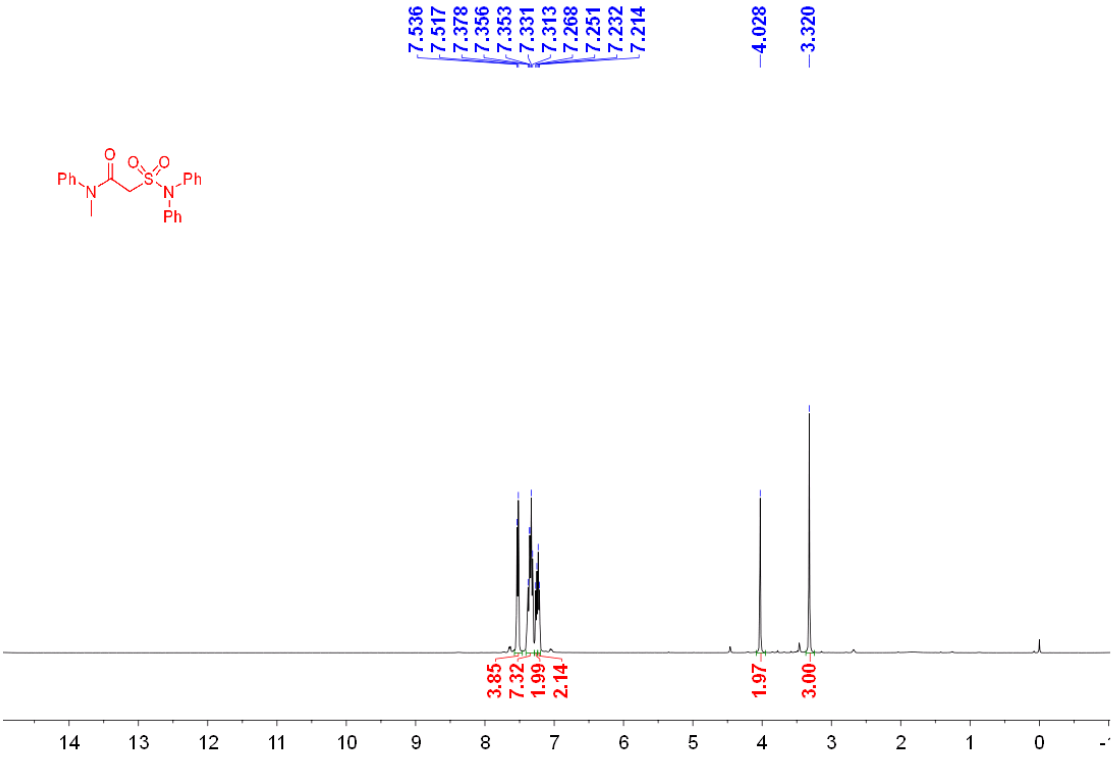

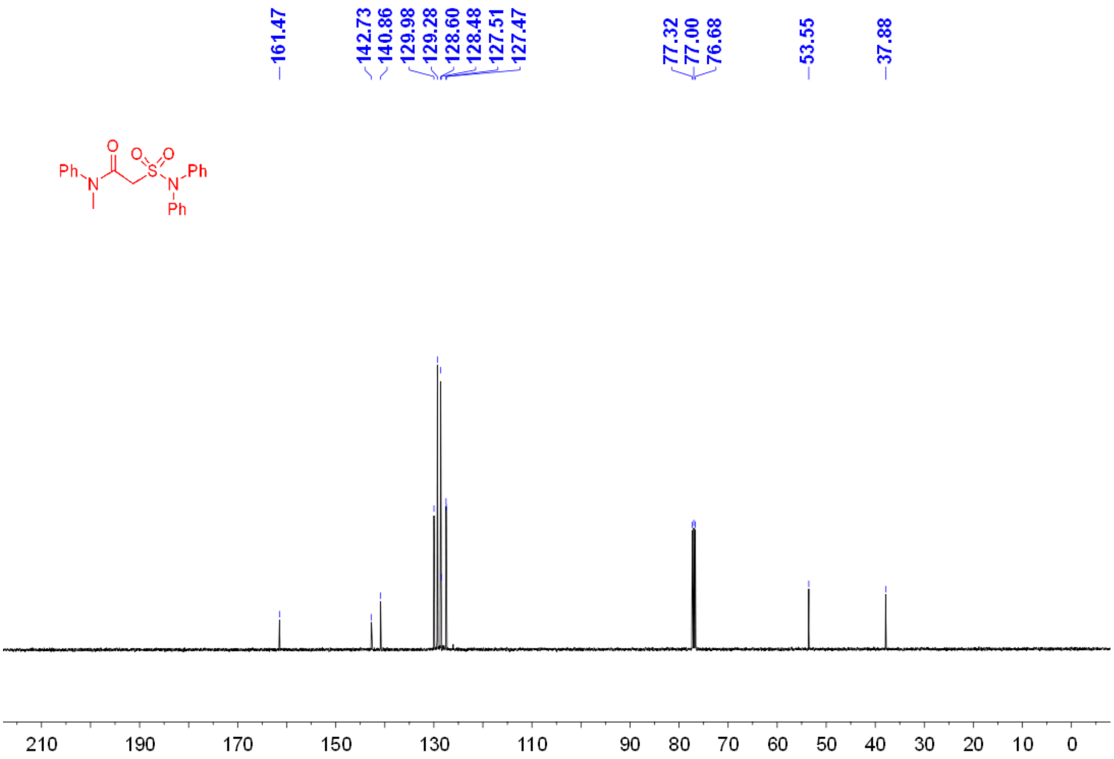

2-(*N*-Methyl-*N*-phenylsulfamoyl)-*N*,*N*-diphenylacetamide (7g):

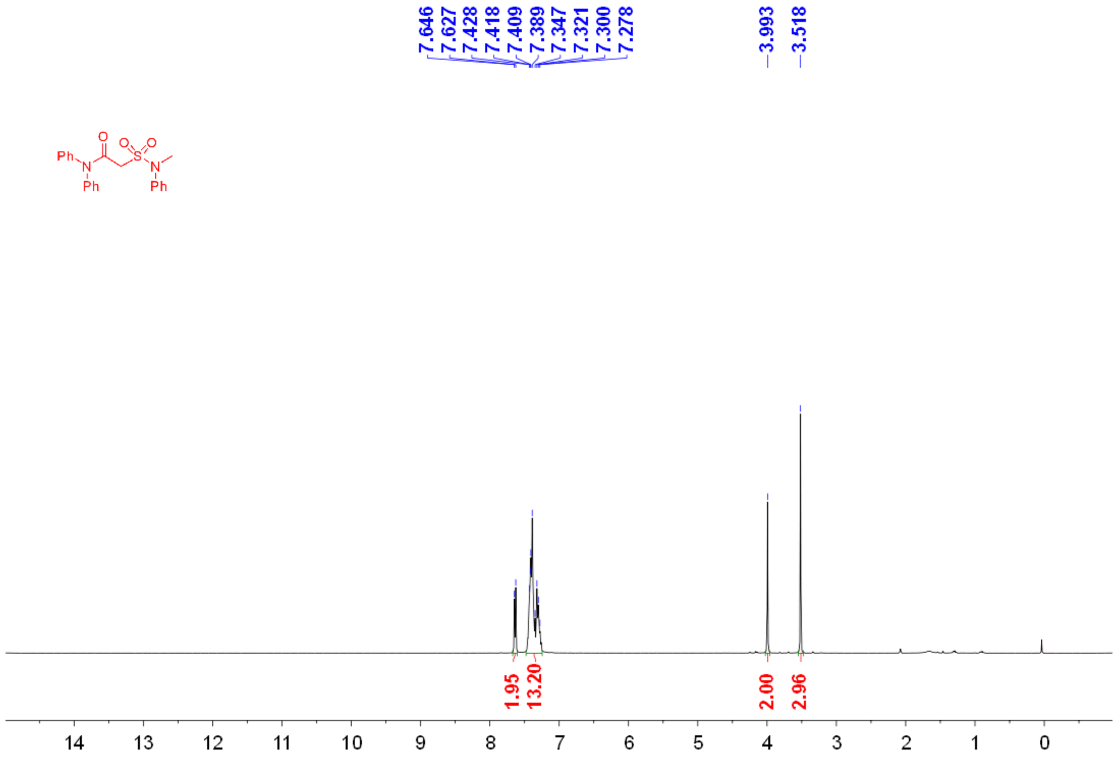

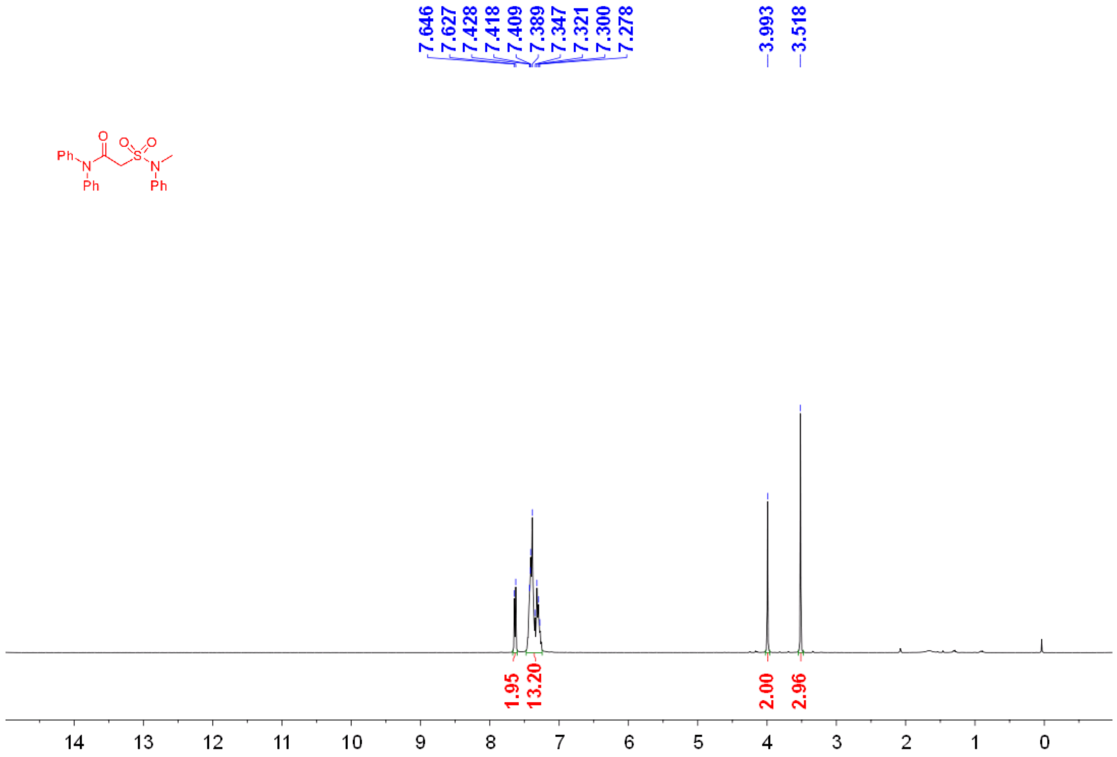

***N*,*N*-Diphenyl-2-(*N*,*N*-diphenylsulfamoyl)acetamide (7h):**

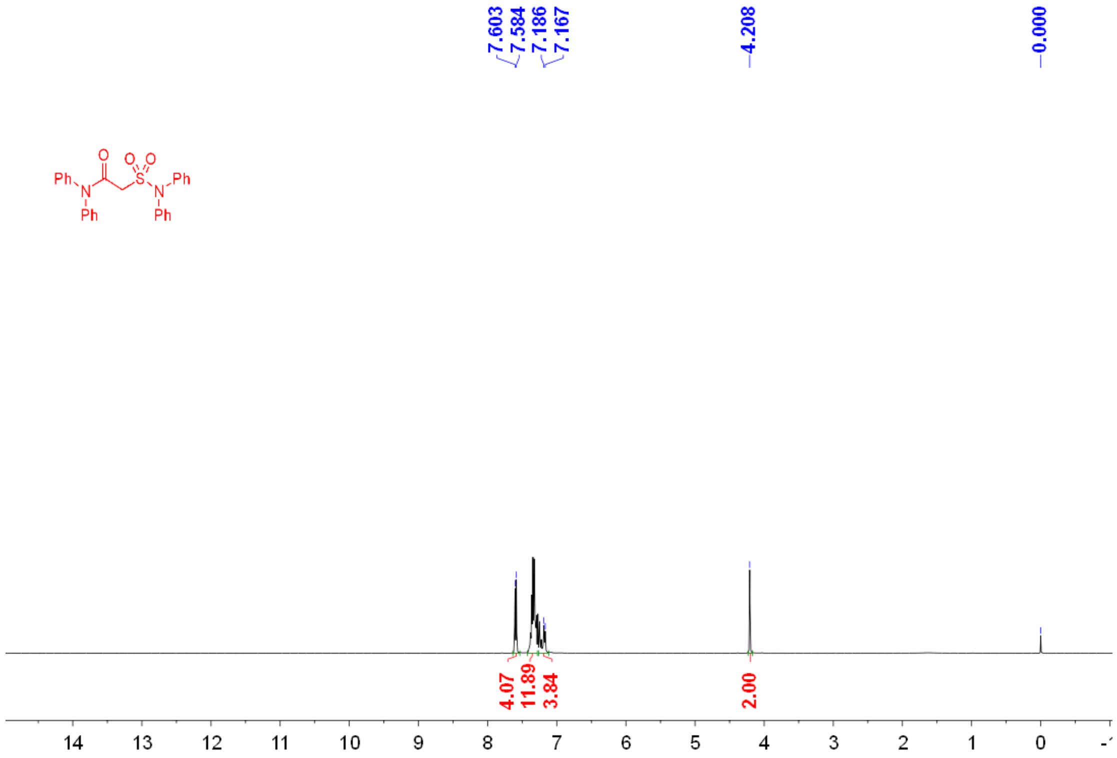

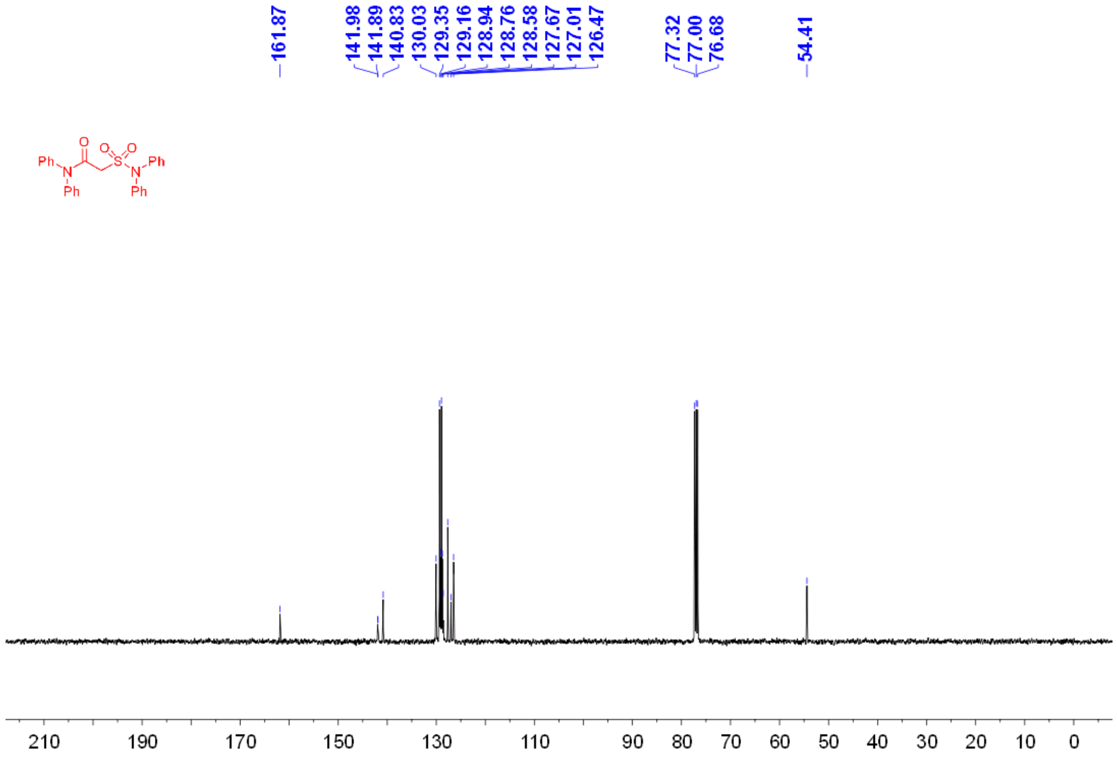

2-(*N*-Benzyl-*N*-butylsulfamoyl)-2-diazo-*N*-methyl-*N*-phenylacetamide (1c)

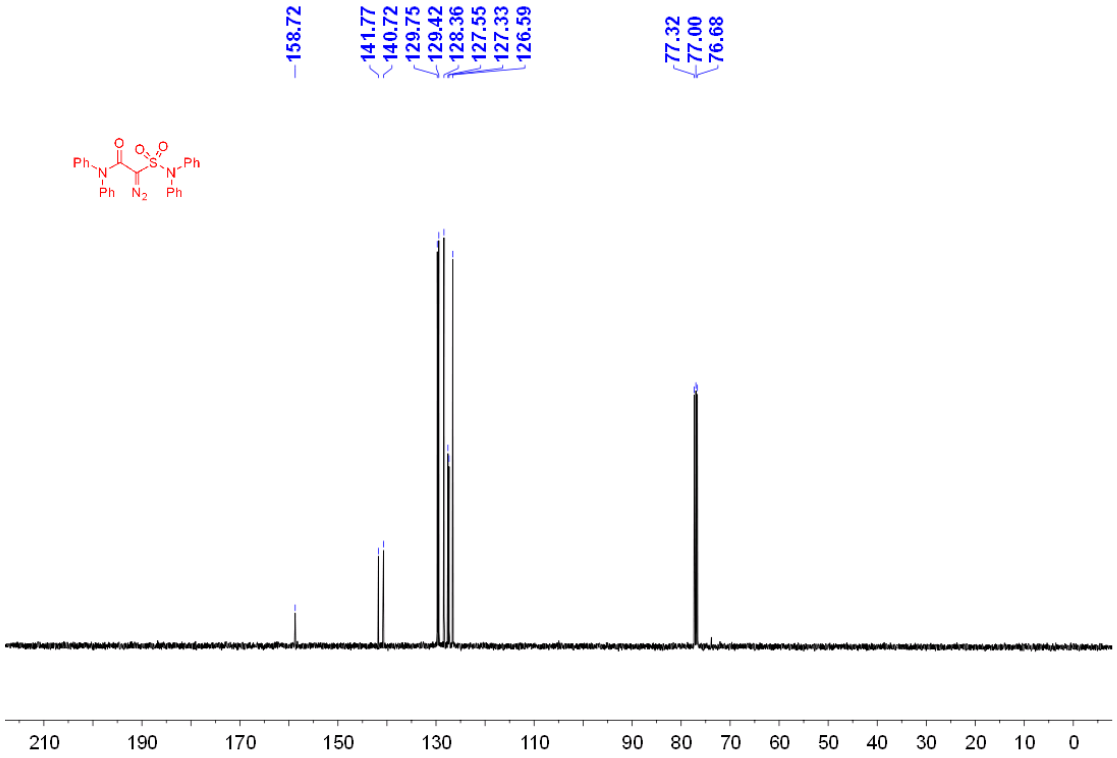

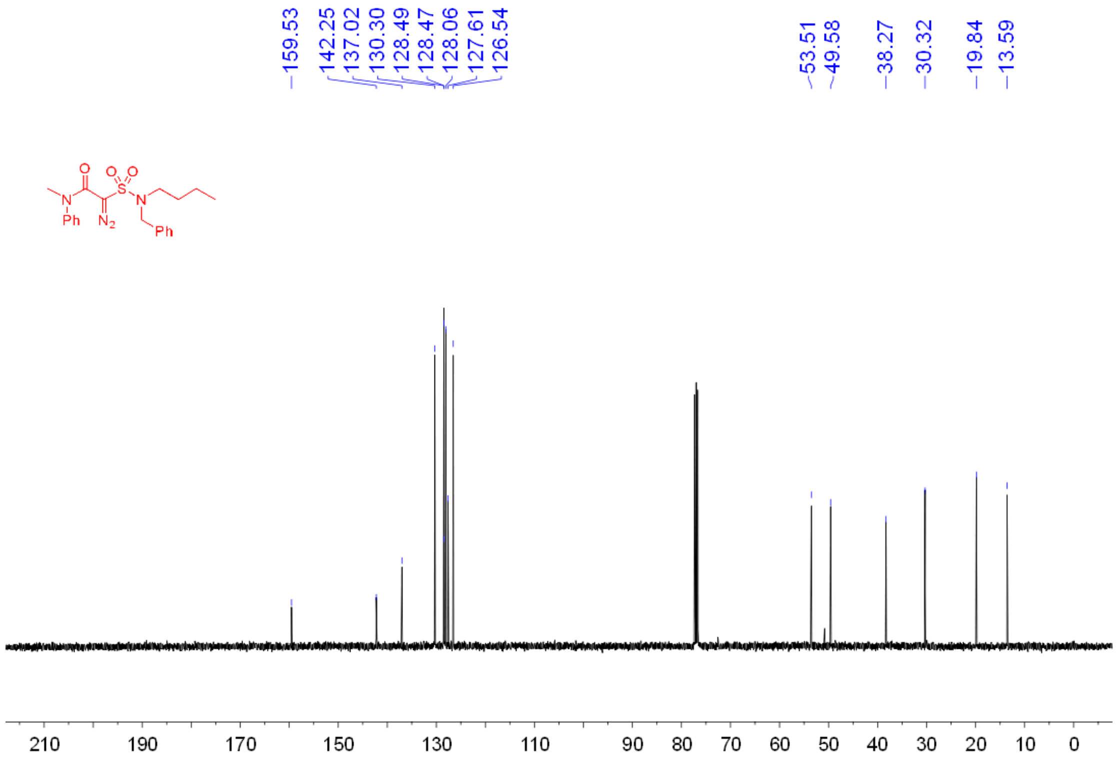

2-(*N*-Benzyl-*N*-butylsulfamoyl)-2-diazo-*N*,*N*-diphenyl acetamide (1d)

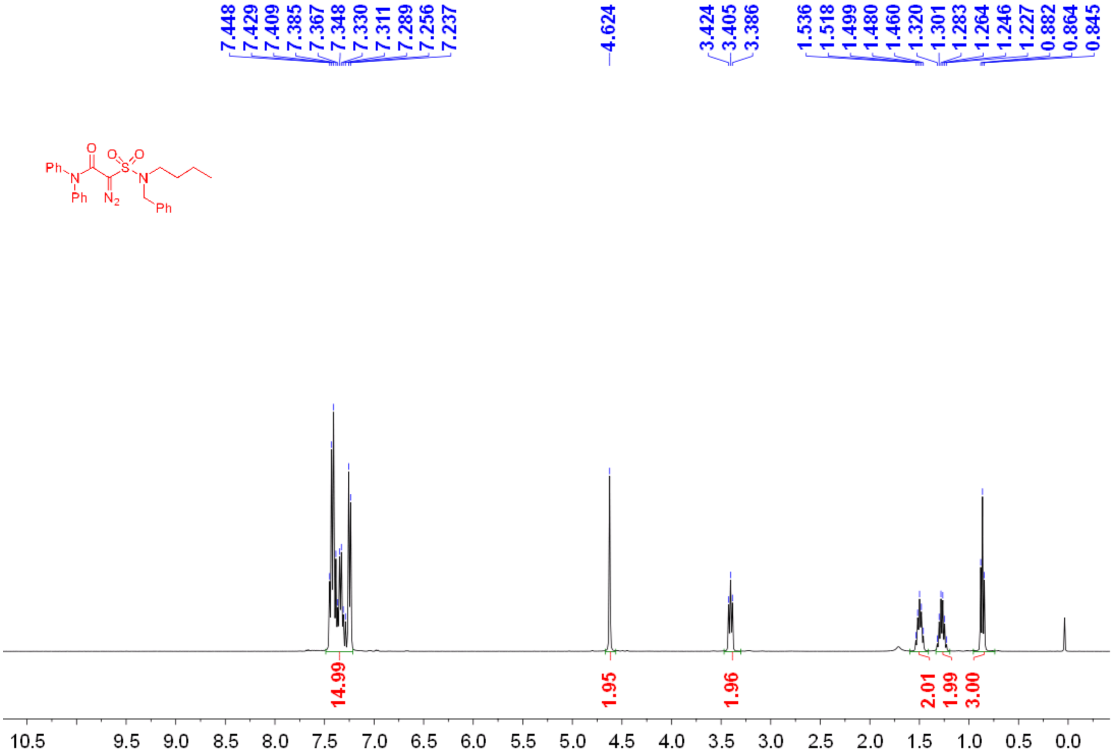

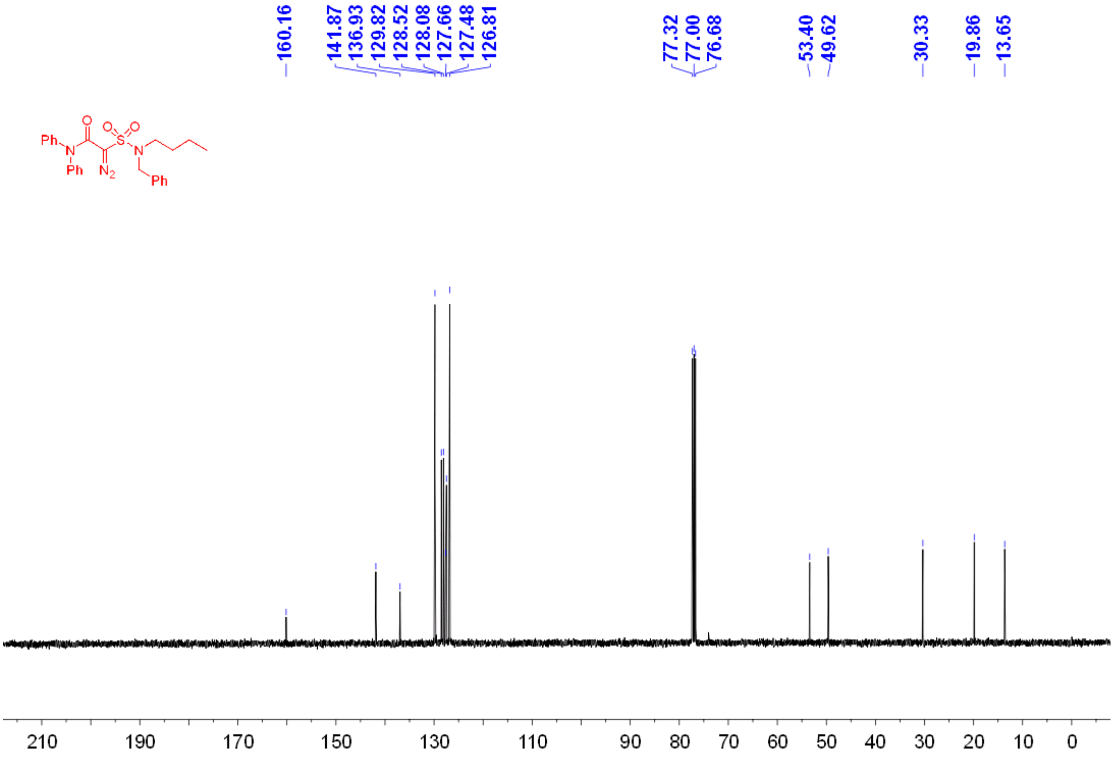

2-Diazo-*N*-methyl-2-(*N*-methyl-*N*-phenylsulfamoyl)-*N*-phenylacetamide (1e):

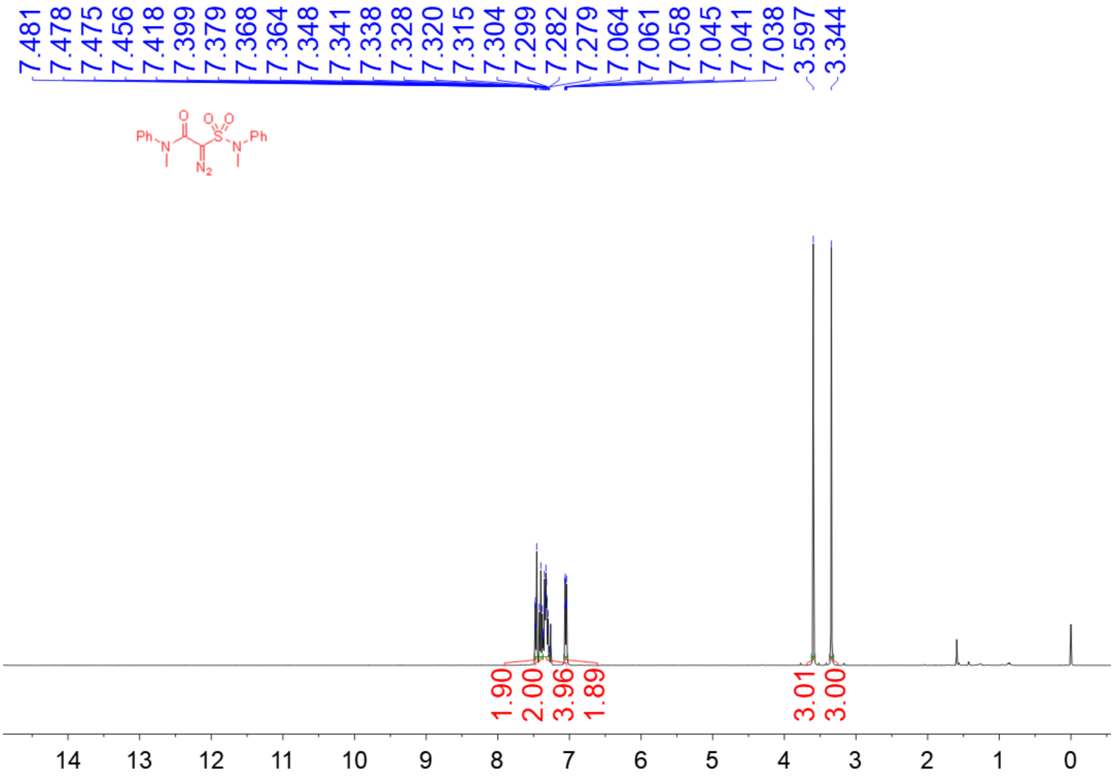

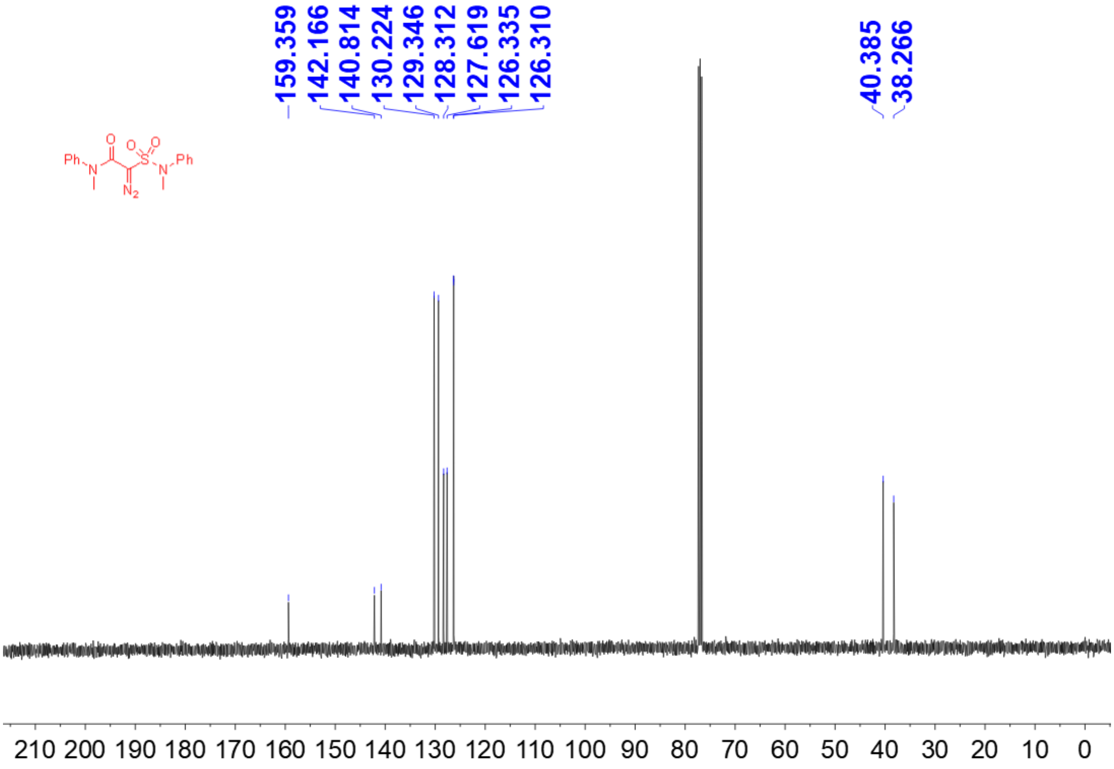

2-Diazo-*N*-methyl-*N*-phenyl-2-(*N*,*N*-diphenylsulfamoyl)acetamide (1f)

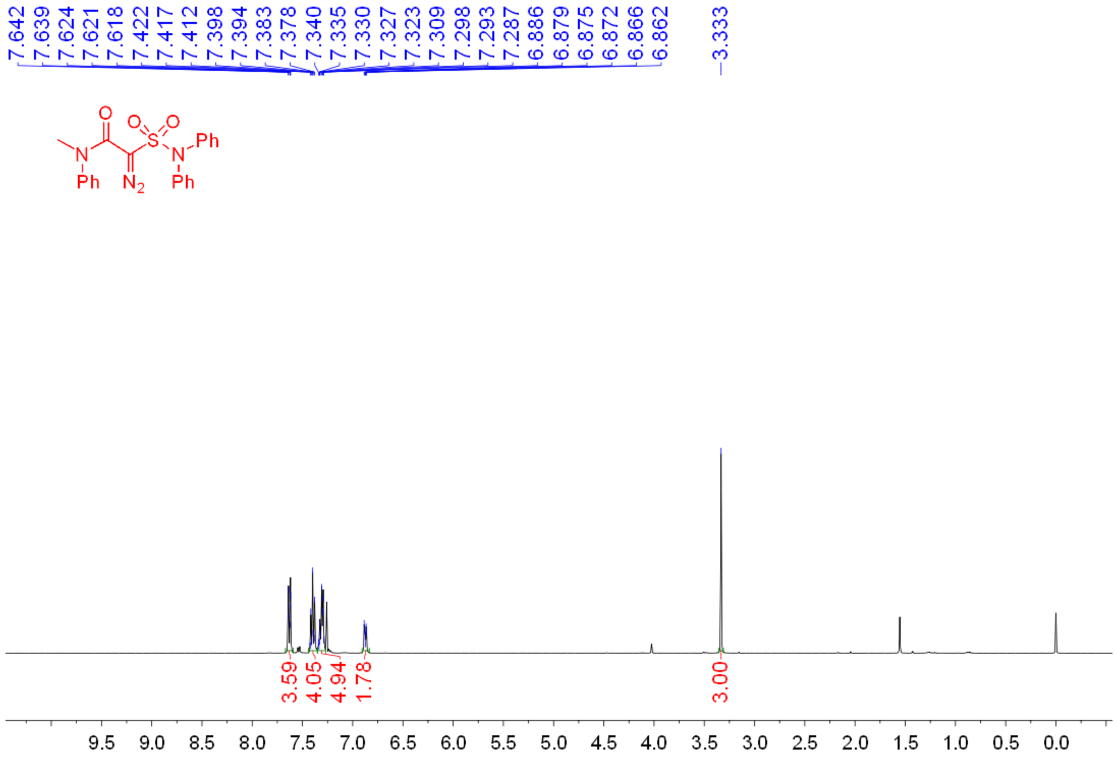

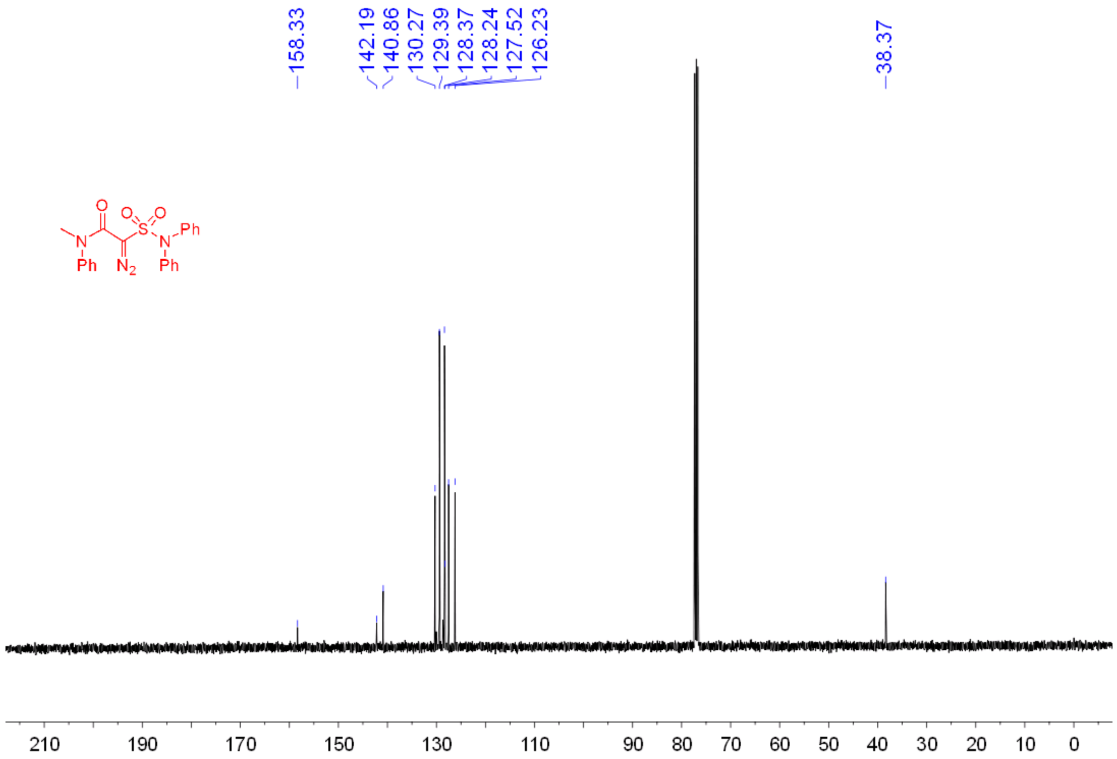

2-Diazo-2-(*N*-methyl-*N*-phenylsulfamoyl)-*N*,*N*-diphenylacetamide (1g):

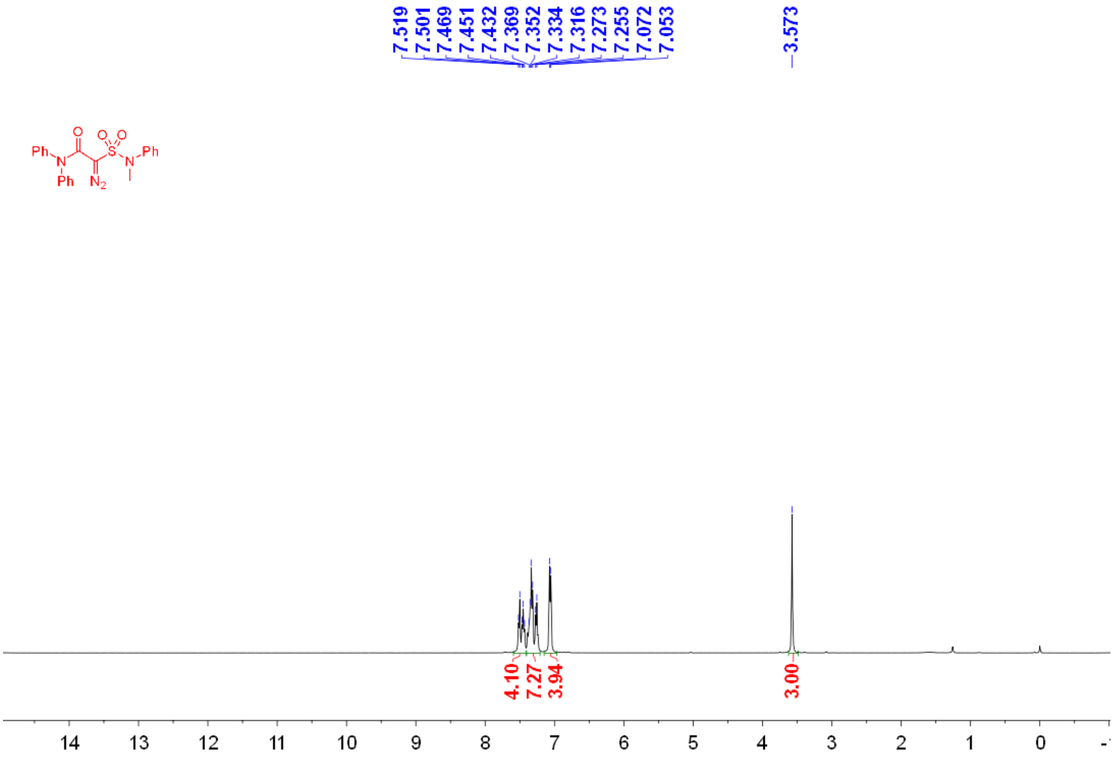

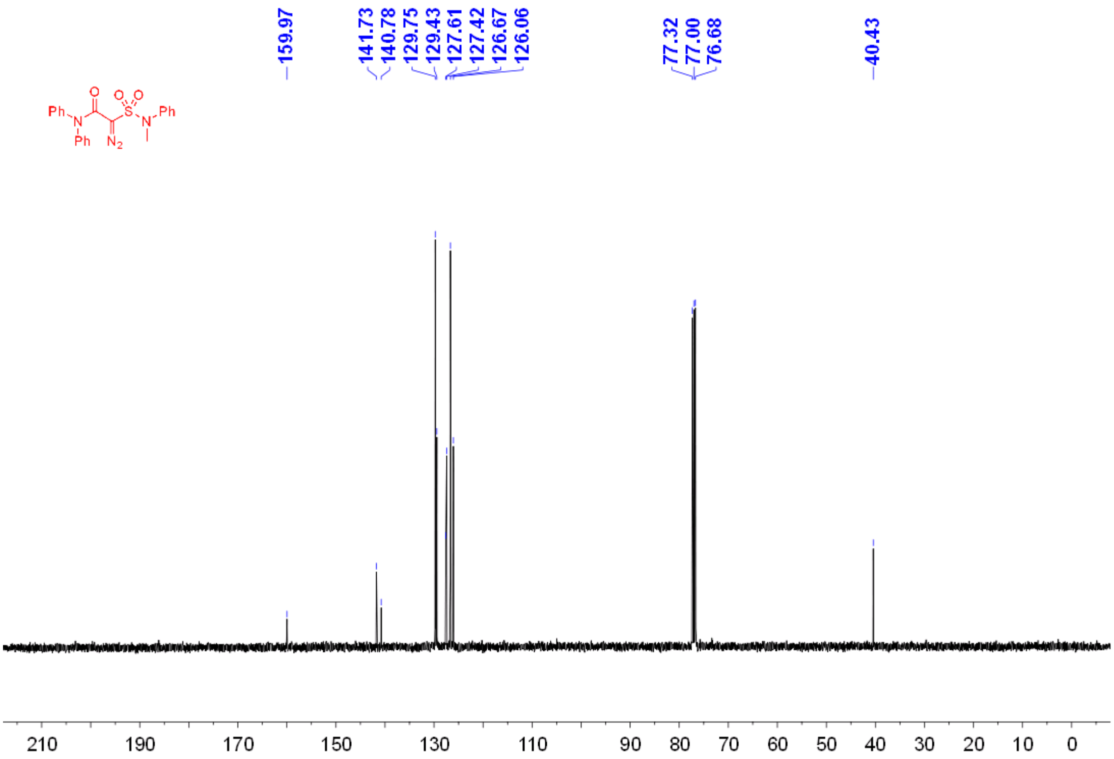

2-Diazo-*N*,*N*-diphenyl-2-(*N*,*N*-diphenylsulfamoyl)acetamide (1h)

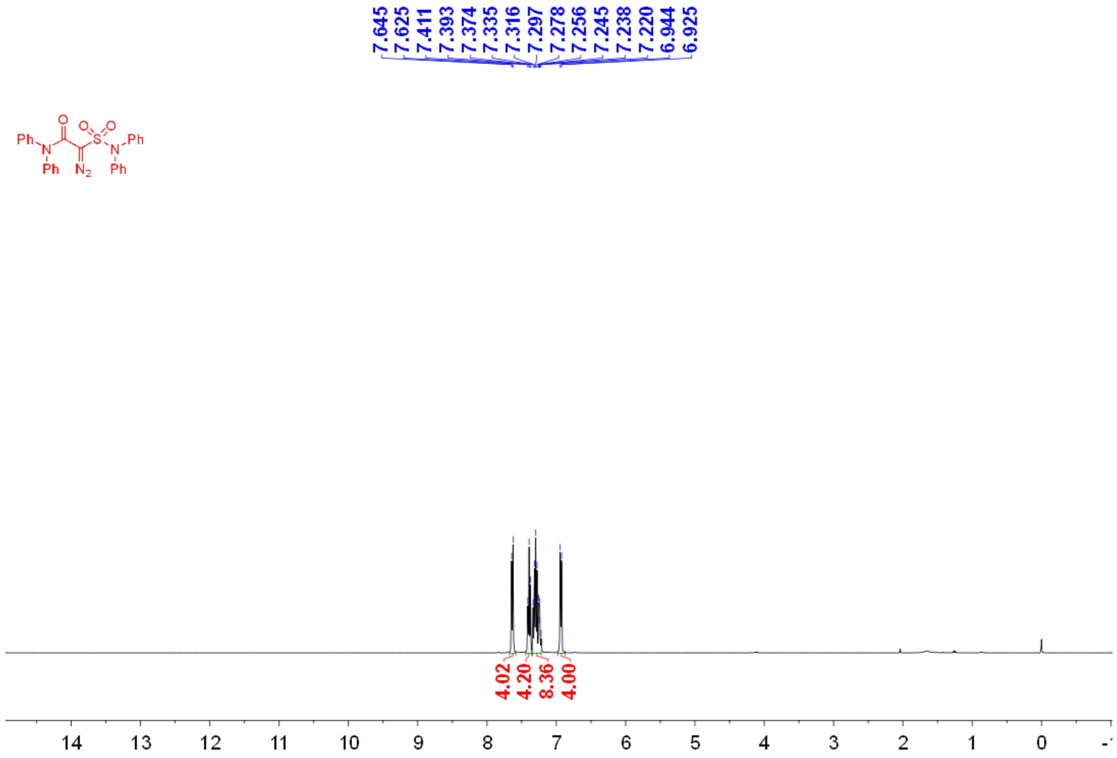

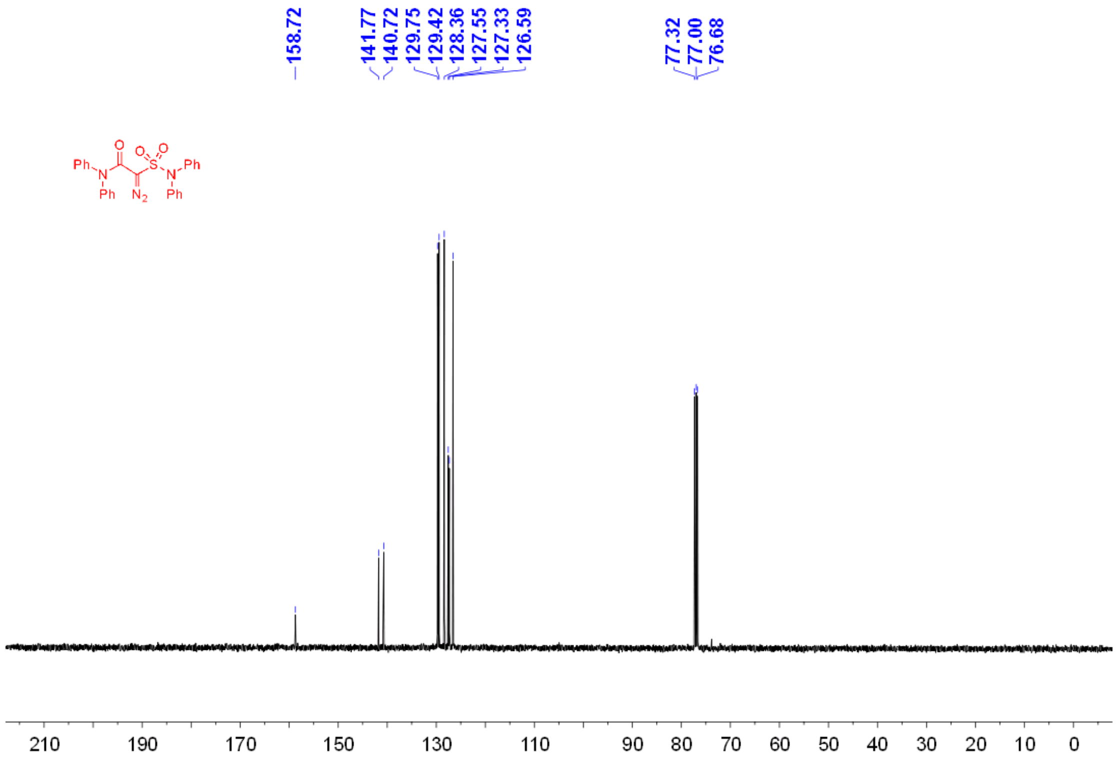

***N*,*N*-Dimethyl-2,2-dioxido-1-phenyl-1,3-dihydrobenzo[*c*]isothiazole-3-carboxamide (2b):**

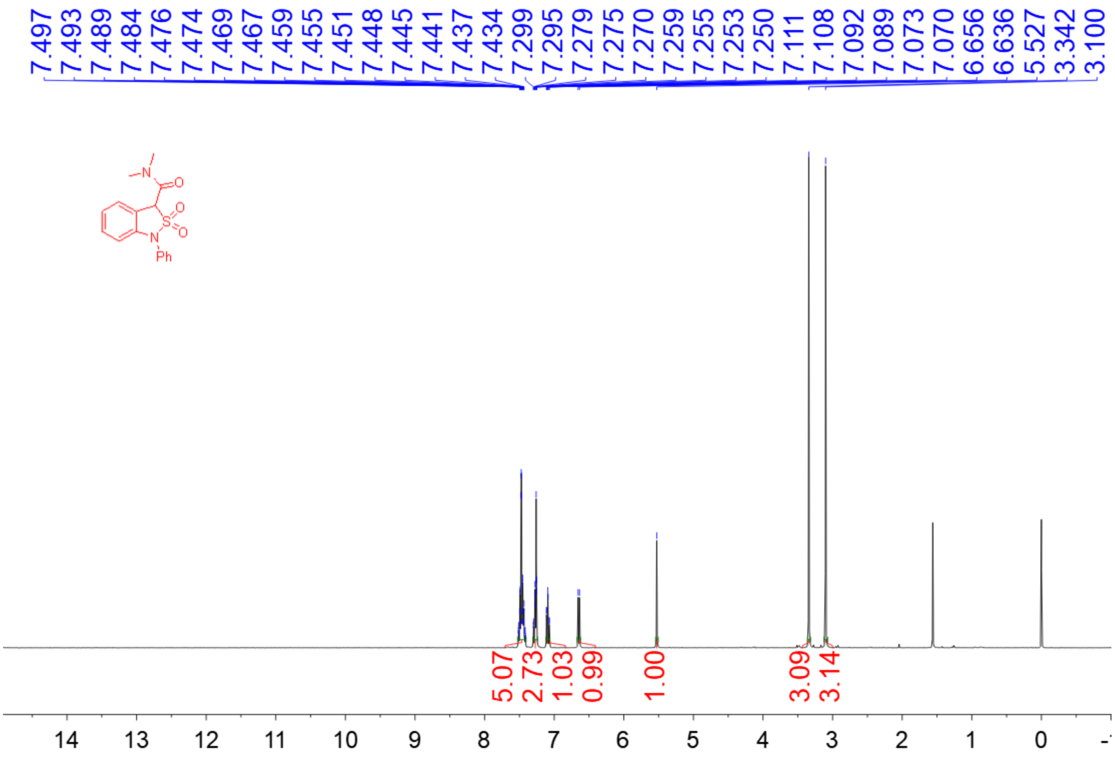

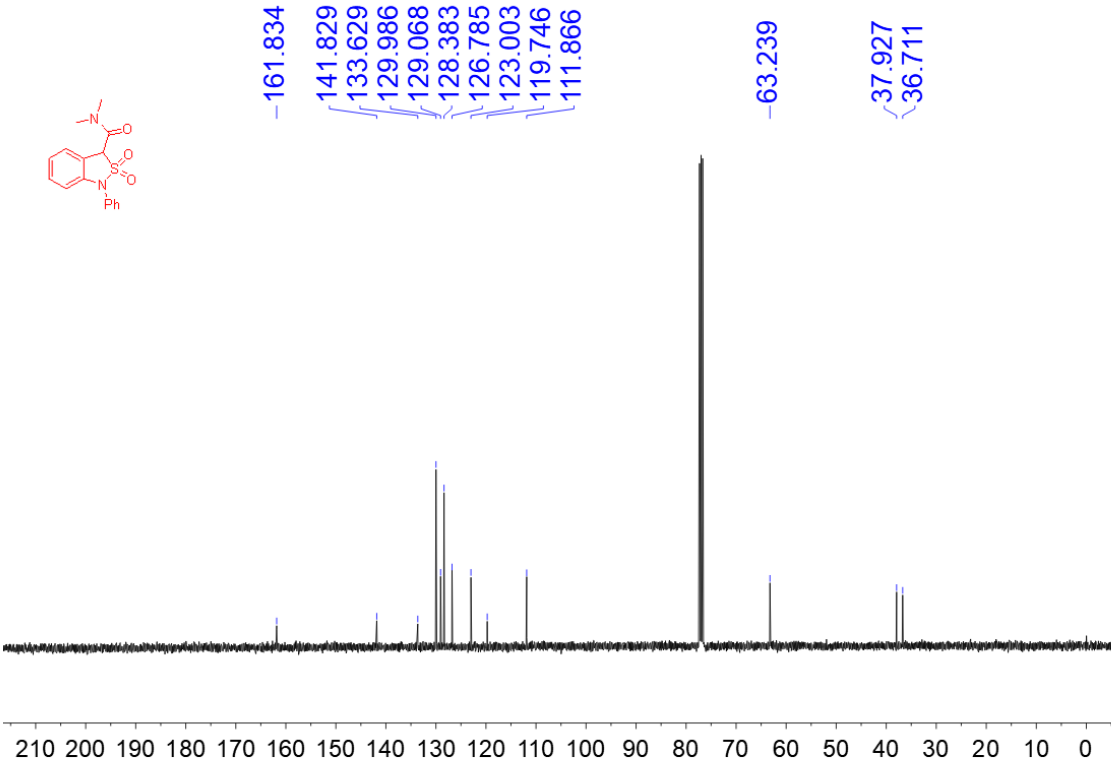

***N*-Benzyl-*N*-butyl-2-oxo-1-methylindoline-3-sulfonamide (3c)**

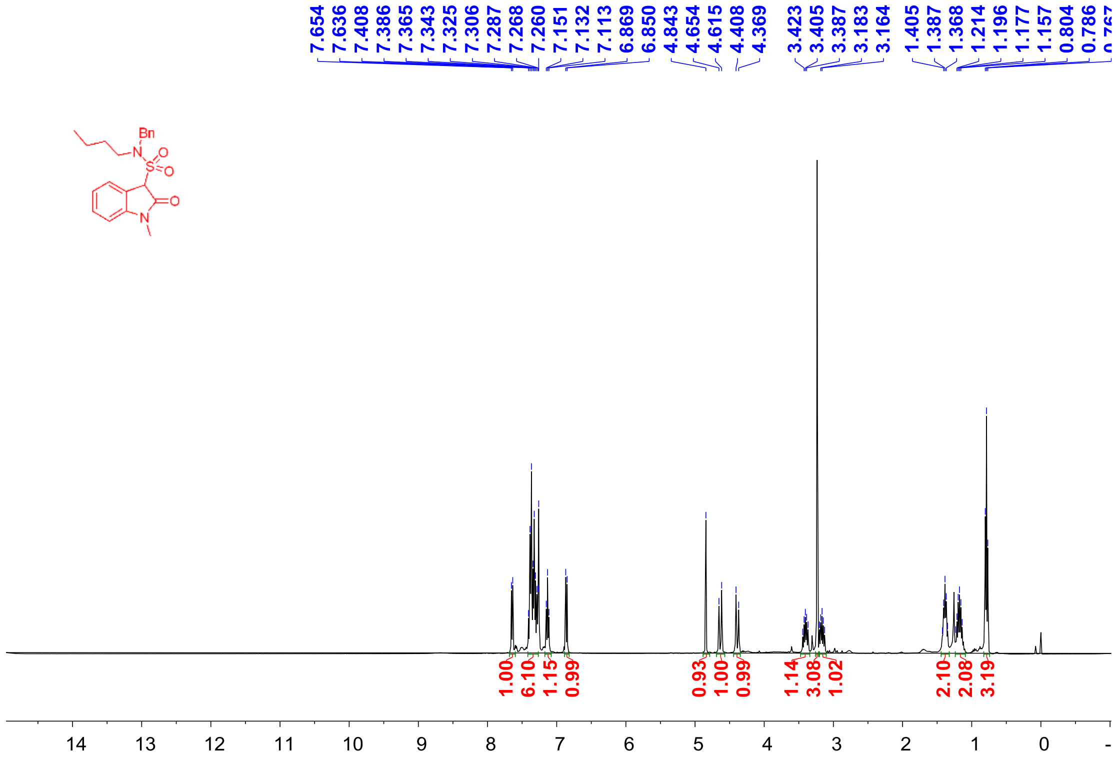

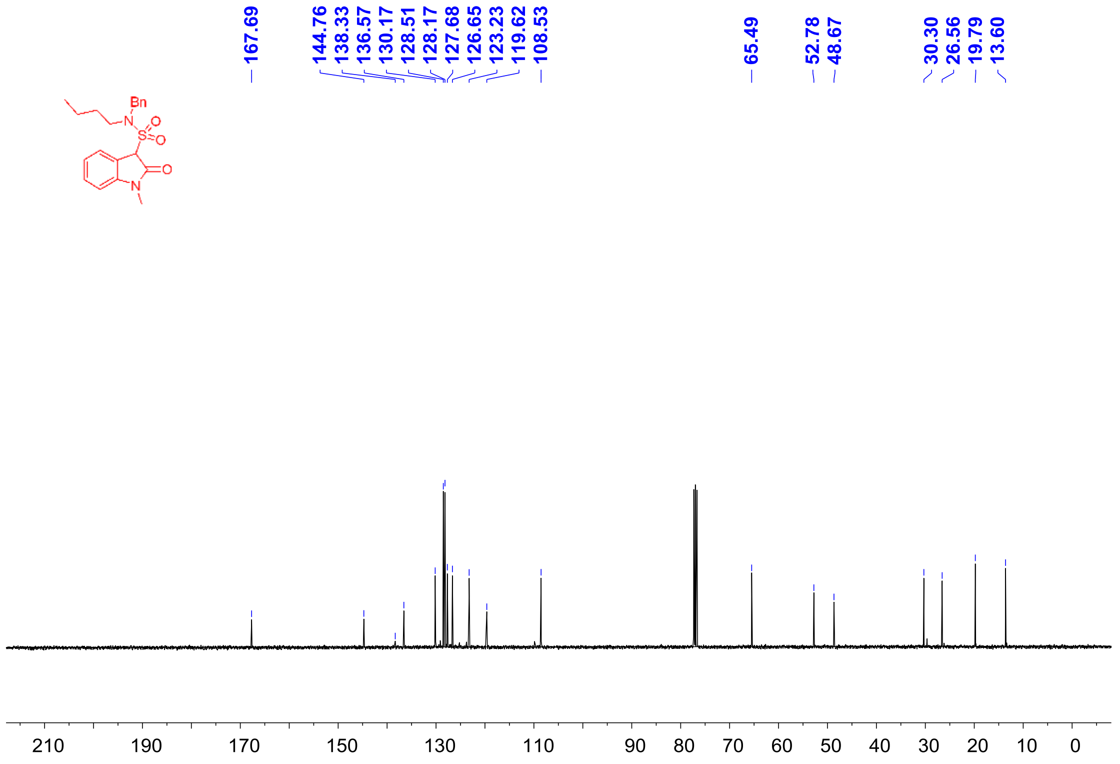

***N*-Benzyl-*N*-butyl-2-oxo-1-phenylindoline-3-sulfonamide (3d)**

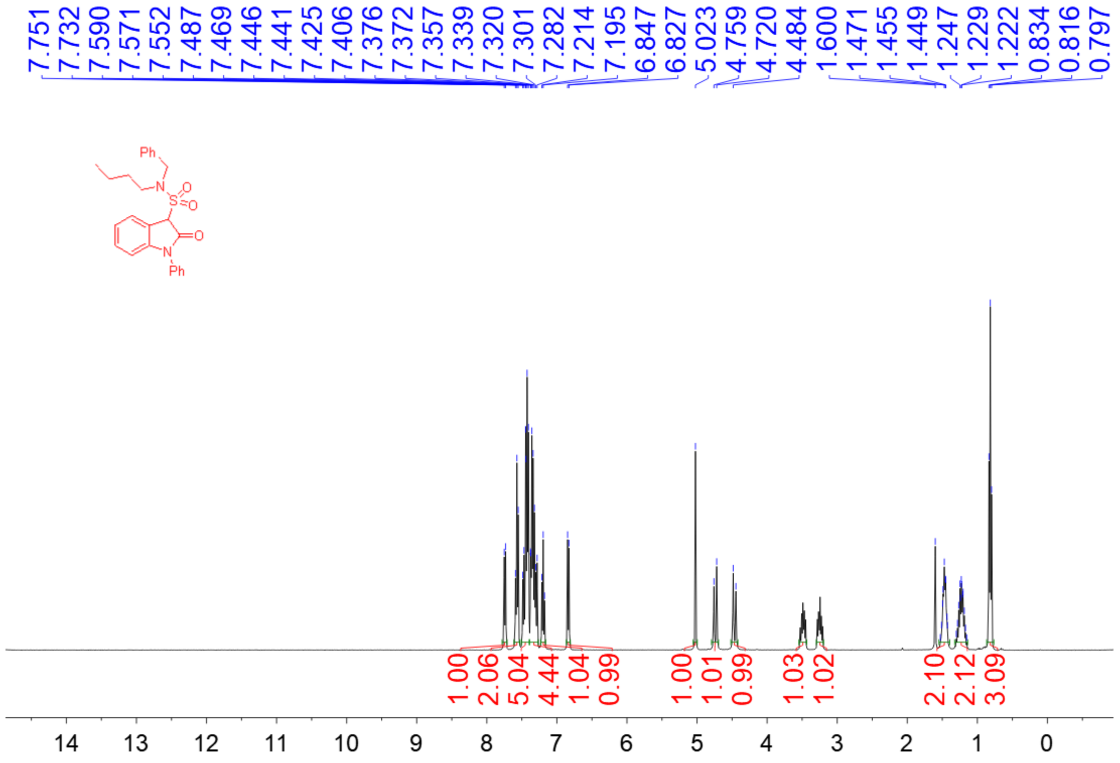

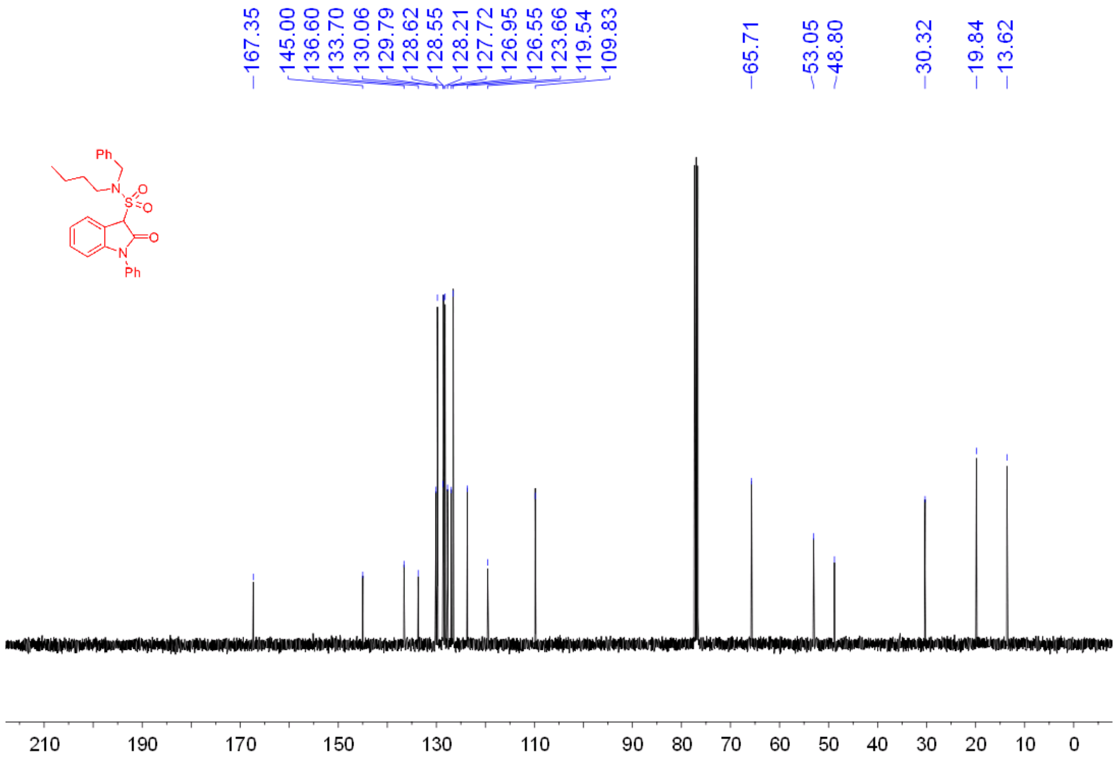

N,1-Dimethyl-2-oxo-N-phenylindoline-3-sulfonamide (3e):

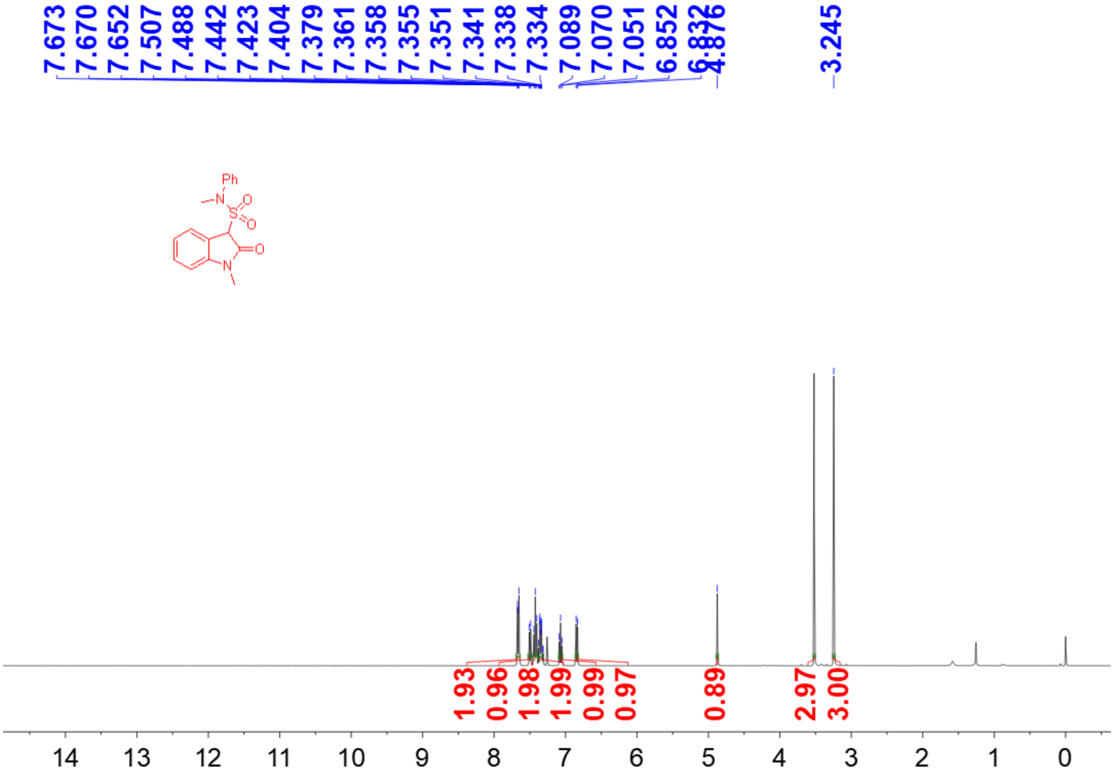

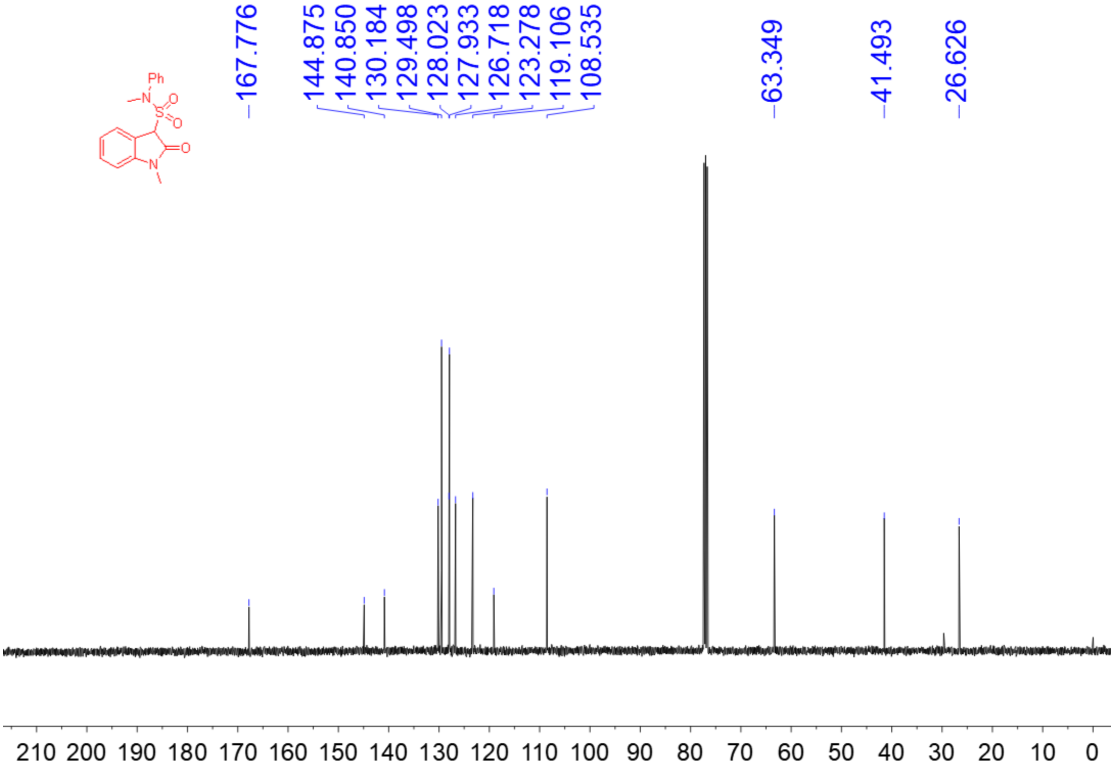

***N*-Methyl-2,2-dioxido-*N*,1-diphenyl-1,3-dihydrobenzo[*c*]isothiazole-3-carboxamide (2f) and 1-methyl-2-oxo-*N*,*N*-diphenylindoline-3-sulfonamide (3f):**

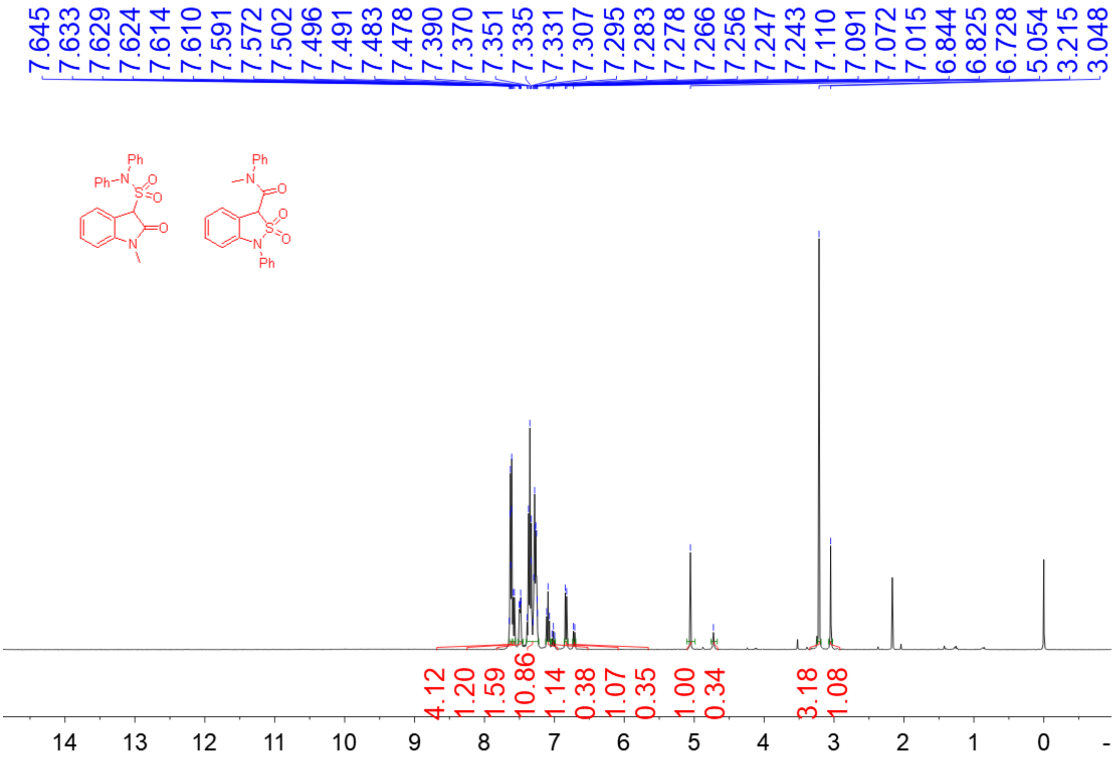

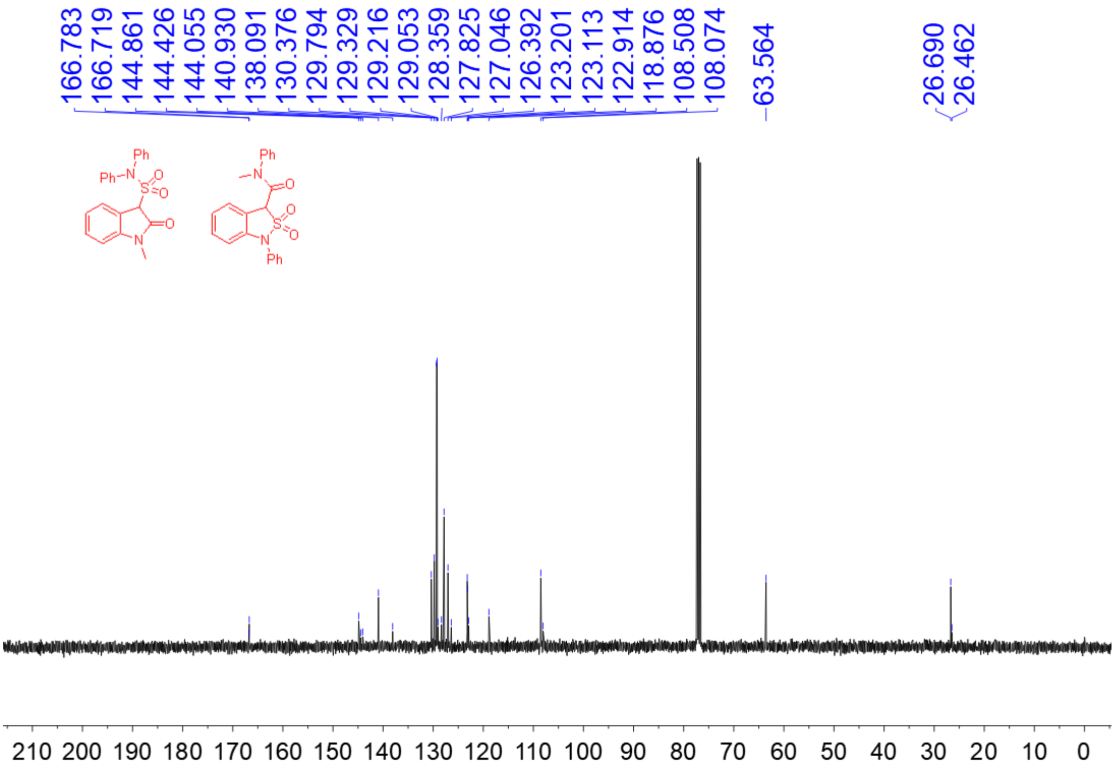

***N*-Methyl-2-oxo-*N*,1-diphenylindoline-3-sulfonamide (3g)**

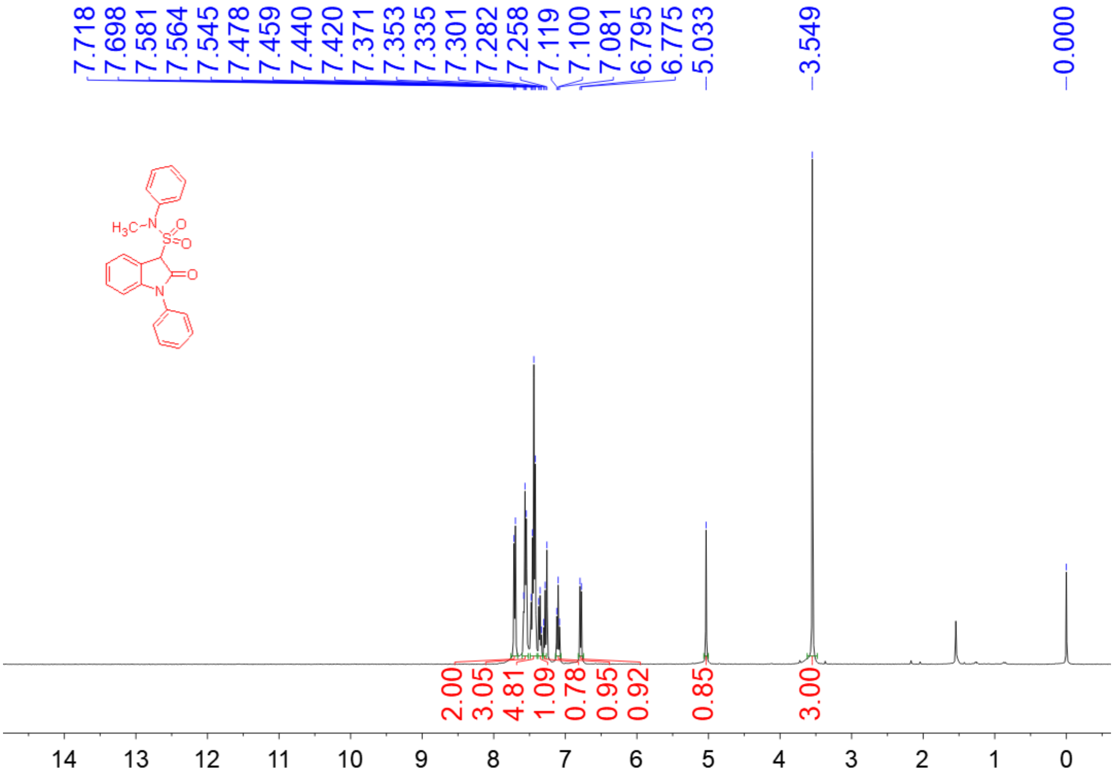

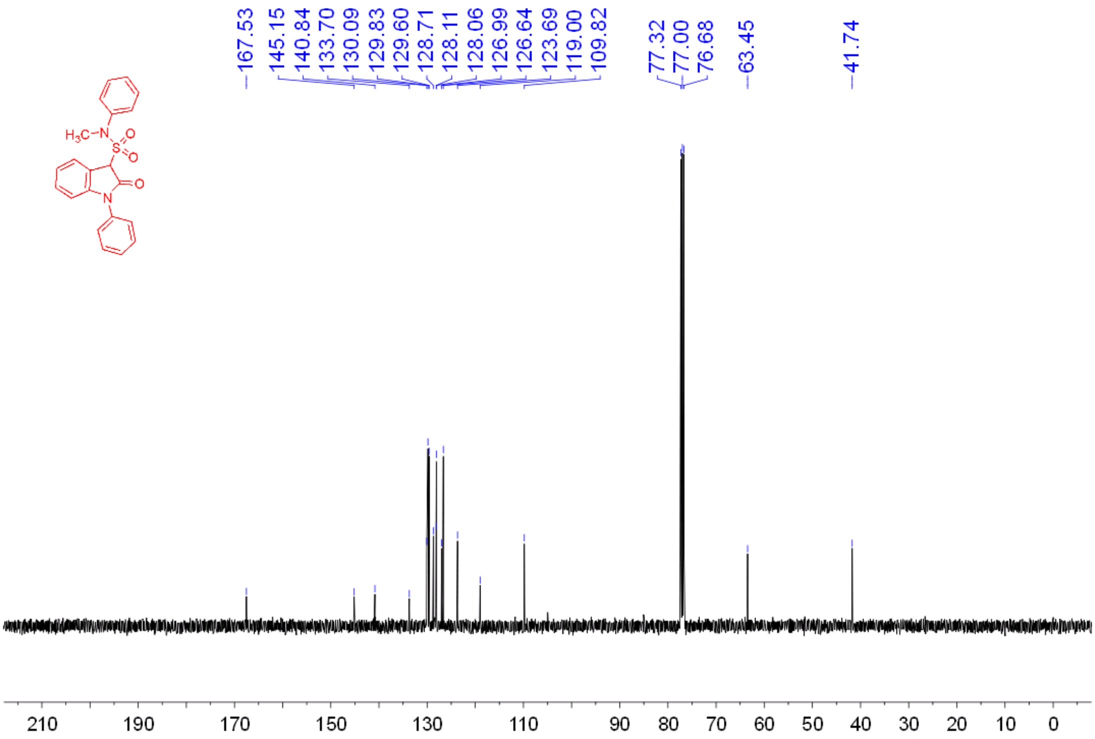

2-Oxo-*N*,*N*,1-triphenylindoline-3-sulfonamide (3h)

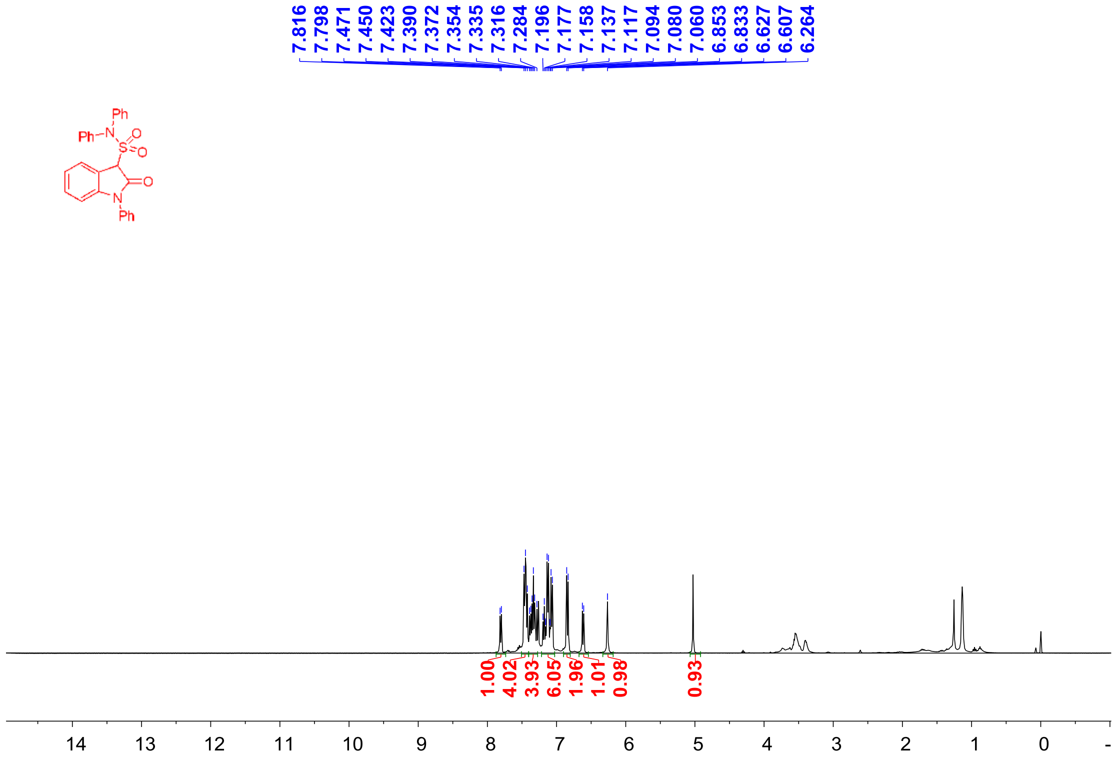

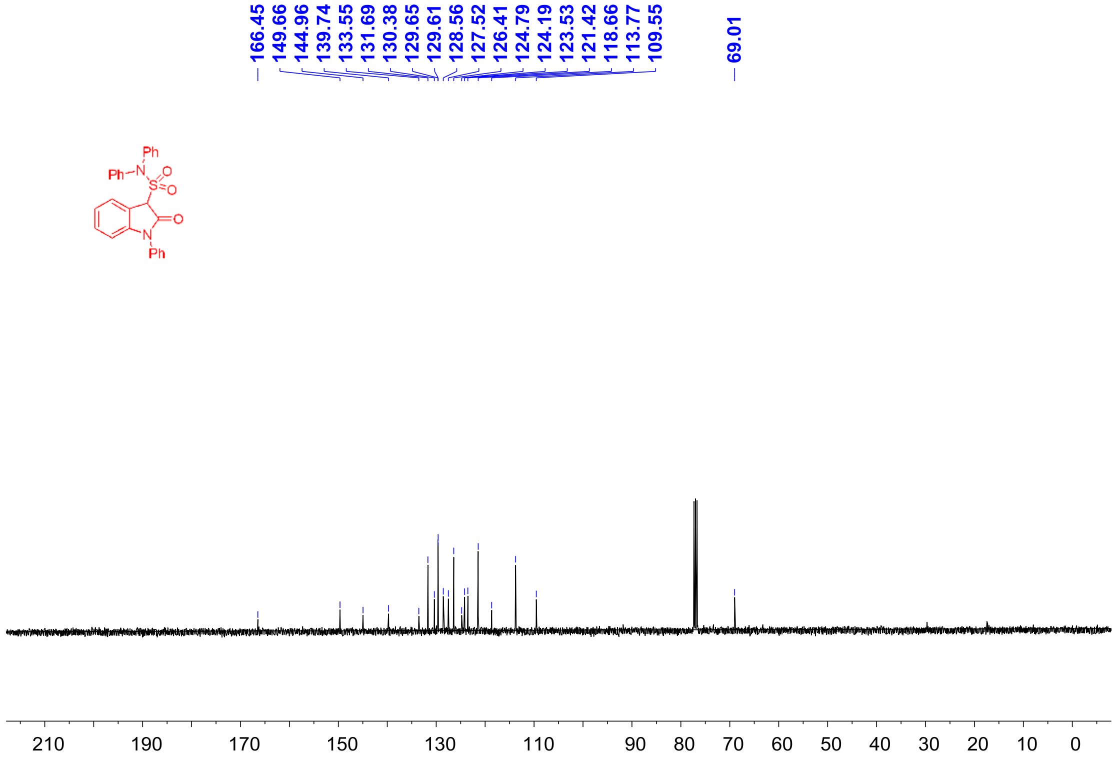

Supplement: Supplementary file 1 [file molecules-24-02628-s001.pdf]
